# Supplementary material for: Learning the bistable cortical dynamics of the sleep-onset period
Source: PLoS Comput Biol. 2026 Apr 30;22(4):e1014246. doi: 10.1371/journal.pcbi.1014246 (PMC13152216; doi:10.1371/journal.pcbi.1014246)
Supplement: S1 Appendix — Text B. Affine link between μ(t) and V2. Fig A. Explained variance of SVD modes in wake and sleep segments. Fig B. The reported association between cubic parameters and subjective sleepiness is not driven by participants who have low model fit. Fig C. Parameter–behavior associations remain consistent across σobs settings. Fig D. Comparison of embeddings with Li et al. (2025). Fig E. First singular modes (U1) of a global SVD analysis of the full SOP spectrogram, for all participants in our test dataset. Fig F. Temporal weights of the first singular modes (V(:,1)T), superimposed to total spectral power. Fig G. Spectrogram localization of SOP windows for all participants. Fig H. Posterior distributions of ϵ. Fig I. Example posterior draws with minimal RMSE. Fig J. Distribution of KL-divergence and RMSE over posterior samples. (PDF) [file pcbi.1014246.s001.pdf]

S1 Appendix

Learning the bistable cortical dynamics of the sleep-onset period
Zhenxing Hu, Manaoj Aravind, Xu Lei, J. Nathan Kutz, Jean-Julien Aucouturier

Text A: Analysis of the timecourse of observation and cubic transition likelihoods within individuals

The ratio of the per-timepoint observation and cubic transition likelihood is informative of how the MCMC procedures attributes events in the experimental data to either σ-driven transitions in the cubic dynamics (i.e. amplitude increments on $\dot{x}_t$), or to σ_{obs}-driven observation noise (i.e. amplitude increments on x_t) that operate outside of the state dynamics. The sideways figure below shows the time series of both (z-scored) observation-noise and transition likelihood, as well as the z-scored difference of observation - transition likelihood, along with the modeled trajectory, for each of the 19 participants in our test dataset.

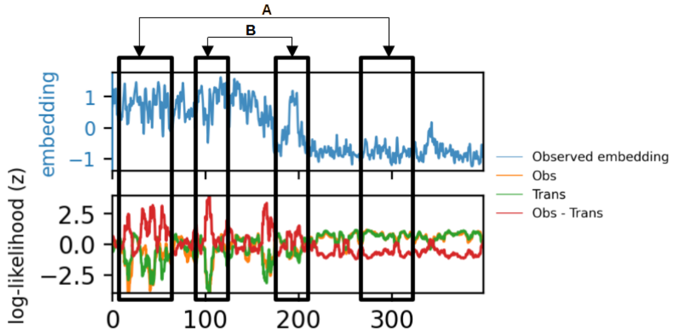

Annotated detail from Subject 01: Time series of z-scored observation-noise (orange), transition likelihood (green), as well as the z-scored difference of observation - transition likelihood (red), along with the modeled embedding (blue).

While the comparison of these time-series is necessarily a bit anecdotal, it appears to confirm the ability of MCMC to trade off noise and model fit: in subject 01 for instance, high noise in the initial part of the embedding is attributed to observation noise, possibly because low noise in the final part has lead to model to adopt a relatively small σ_{cubic} (comparison A); large transitions of comparable amplitudes may be attributed to observation noise or to the dynamics (comparison B) depending on e.g. their temporal position along the sigmoidal change of landscape.

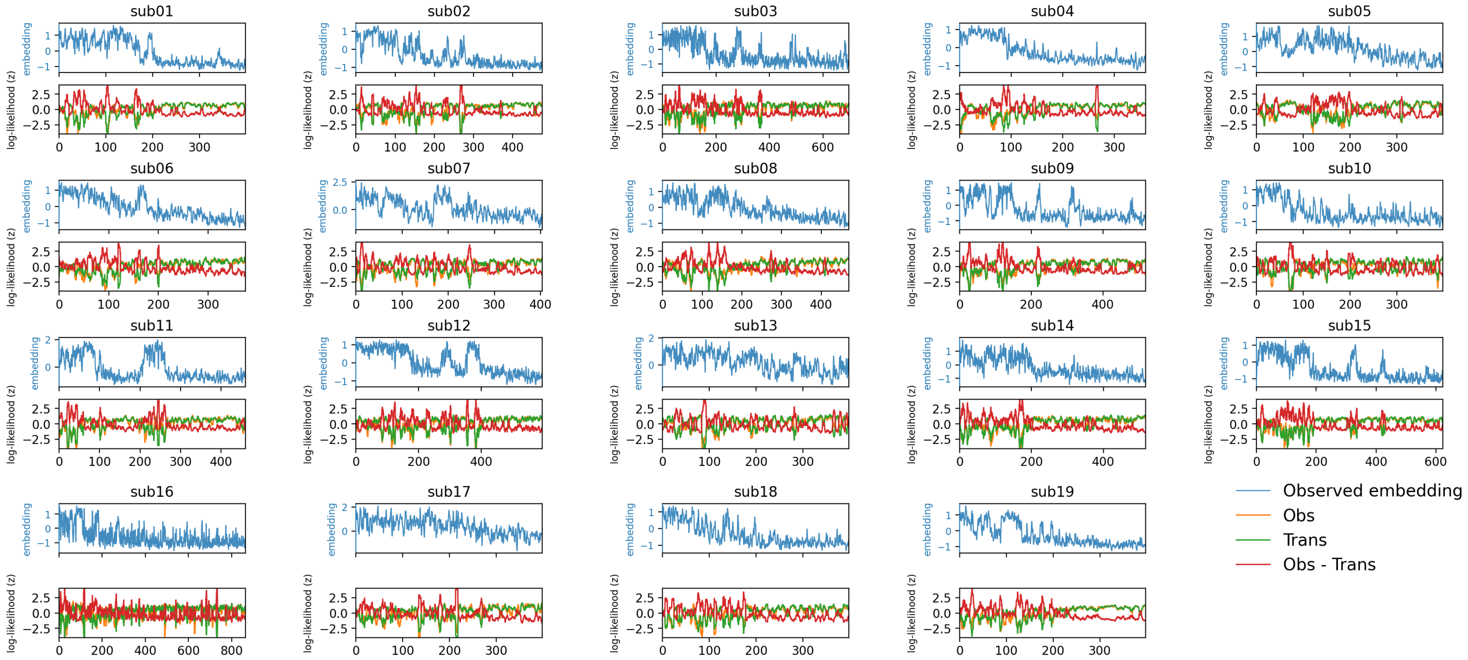

The ratio of the per-timepoint observation and cubic transition likelihood is informative of how the MCMC procedures attributes events in the experimental data to either cubic dynamics or observation noise. Time series of z-scored observation-noise (orange), transition likelihood (green), as well as the z-scored difference of observation - transition likelihood (red), along with the modeled embedding (blue), for each of the 19 participants in our test dataset.

Text B: Affine link between $\mu(t)$ and V_2

Notation and dimensions.

- $M \in \mathbb{R}^{F\times T}$ — raw spectrogram (F frequency bins, T time points);
- $D \in \mathbb{R}^{T\times T}$ — diagonal, $D_{tt} = \|M_{\cdot,t}\|_2^{-1}$ (each column of M is rescaled to unit ℓ_2 norm);
- $\widetilde{M} := MD \in \mathbb{R}^{F\times T}$ — *normalised* spectrogram;
- $U \in \mathbb{R}^{F\times F}$, $V \in \mathbb{R}^{T\times T}$, $\Sigma = \mathrm{diag}(\sigma_1, \sigma_2, \dots)$ — singular-value decomposition (SVD) of the *raw* matrix M:

$$M = U\,\Sigma\,V^\top. \tag{S1}$$

- $U_{\mathrm{w}}^{(1)}, U_{\mathrm{s}}^{(1)} \in \mathbb{R}^{F\times 1}$ — first left singular vectors estimated on wake-only and sleep-only segments;
- $\Delta u := U_{\mathrm{w}}^{(1)} - U_{\mathrm{s}}^{(1)} \in \mathbb{R}^{F\times 1}$.

Projection that defines $\mu(t)$. For every column of the *normalised* spectrogram we project onto the direction Δu:

$$\mu(t) \;=\; \frac{\big\langle\, \widetilde{M}_{\cdot,t} - U_{\mathrm{s}}^{(1)},\ \Delta u \big\rangle}{\|\Delta u\|_2^2}, \qquad t = 1, \dots, T. \tag{S2}$$

Gathering the scalars into a row vector $\mu^\top = [\mu(1), \dots, \mu(T)] \in \mathbb{R}^{1\times T}$ gives

$$\boxed{\mu^\top \;=\; \frac{\Delta u^\top M D}{\|\Delta u\|_2^2} - \frac{\Delta u^\top U_{\mathrm{s}}^{(1)}}{\|\Delta u\|_2^2}\mathbf{1}^\top}. \tag{S3}$$

Empirical rank-2 structure. Fig **Comparison between SVD modes** shows that the second frequency mode of M coincides with Δu for most subjects:

$$\Delta u \;\approx\; U_2, \qquad \text{where } U_2 \text{ is the second left singular vectors of } U. \tag{S4}$$

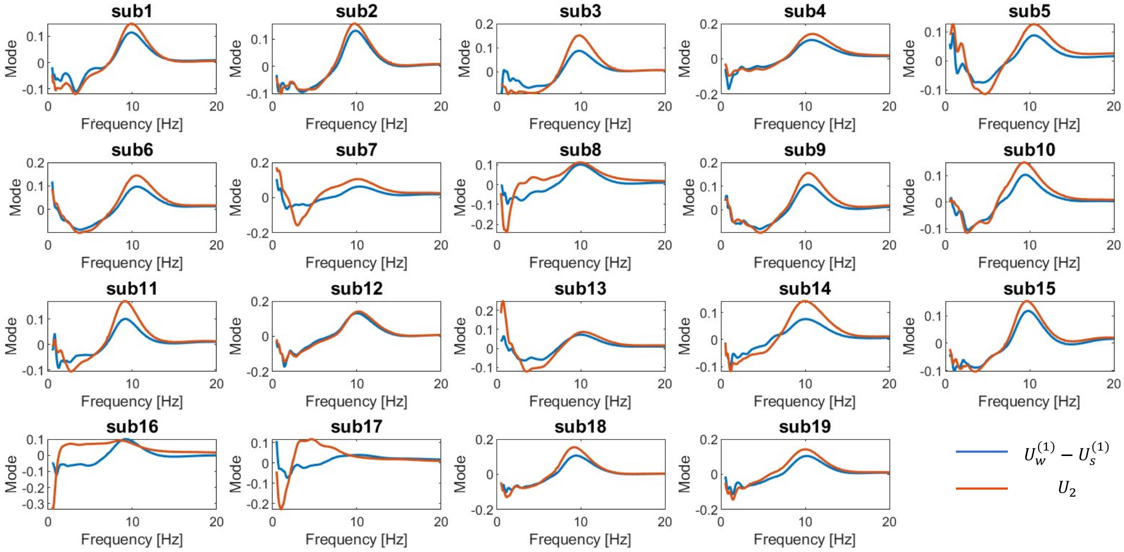

Comparison between SVD modes. For every participant, the difference between the first left singular vectors extracted from wake-only ($U_w^{(1)}$) and sleep-only ($U_s^{(1)}$) spectrogram blocks (blue) closely matches the second left singular vector of the full raw spectrogram, U_2 (orange).

Action of $U_2^\top$ on the MD product. Left-multiplying (S1) by $U_2^\top$ and right-multiplying by D yields

$$U_2^\top M D \;=\; U_2^\top U\,\Sigma\,V^\top D \;=\; \sigma_2\,V_2^\top D, \tag{S5}$$

Affine relation between μ and V_2. Substituting (S4) into (S3) and then invoking (S5) gives the *explicit* affine map

$$\boxed{\mu^\top \;\approx\; \underbrace{\frac{\sigma_2}{\|\Delta u\|_2^2}}\,V_2^\top D \;+\; \underbrace{\left(-\frac{\Delta u^\top U_{\mathrm{s}}^{(1)}}{\|\Delta u\|_2^2}\right)}\,\mathbf{1}^\top}\,, \tag{S6}$$

Equation (S6) confirms that the one-dimensional embedding $\mu(t)$ and the right singular vector V_2 (after the column-wise normalisation encoded by D) contain the *same* information up to a fixed scaling a and offset b.

S1 Figures:

Fig A: Explained variance of SVD modes in wake and sleep segments
Fig B: The reported association between cubic parameters and subjective sleepiness is not driven by participants who have low model fit
Fig C: Parameter–behavior associations remain consistent across σ_{obs} settings
Fig D: Comparison of embeddings with Li et al. (2025)
Fig E: First singular modes (U^1) of a global SVD analysis of the full SOP spectrogram, for all participants in our test dataset.
Fig F: Temporal weights of the first singular modes ($V^T_{(:,1)}$), superimposed to total spectral power
Fig G: Spectrogram localization of SOP windows for all participants.
Fig H: Posterior distributions of ϵ
Fig I: Example posterior draws with minimal RMSE.
Fig J: Distribution of KL-divergence and RMSE over posterior samples.

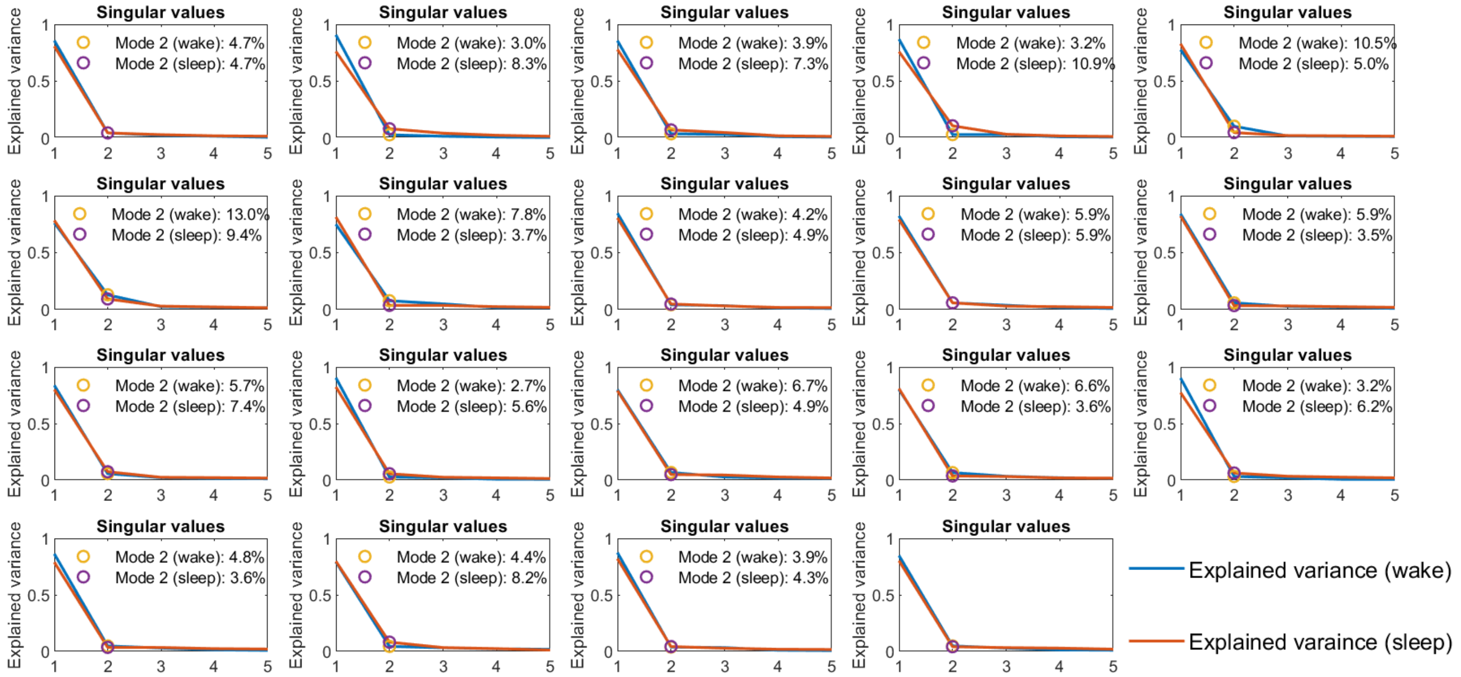

Fig A: Explained variance of SVD modes in wake and sleep segments.
For each participant, we compute the fraction of variance explained by the leading SVD modes separately within the wake (blue) and sleep (orange) spectrogram segments used to construct $\mu(t)$. Across subjects, there is a pronounced rank-1 structure for the wake and sleep segments, with the second mode accounting for only a small additional fraction of variance.

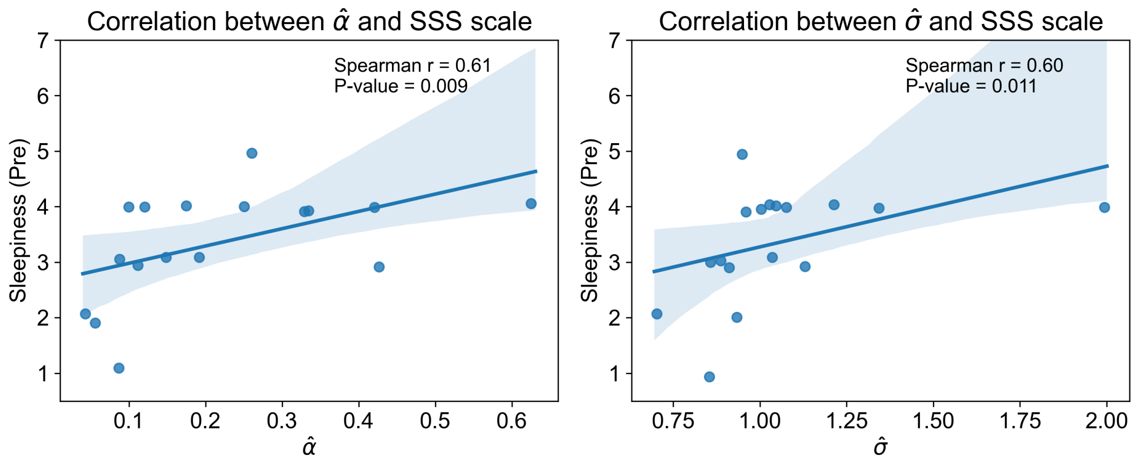

Fig B: The reported association between cubic parameters and subjective sleepiness is not driven by participants who have low model fit. Regression plots excluding the 10%-percentile of mode of transition likelihood (subjects 13 and 17)

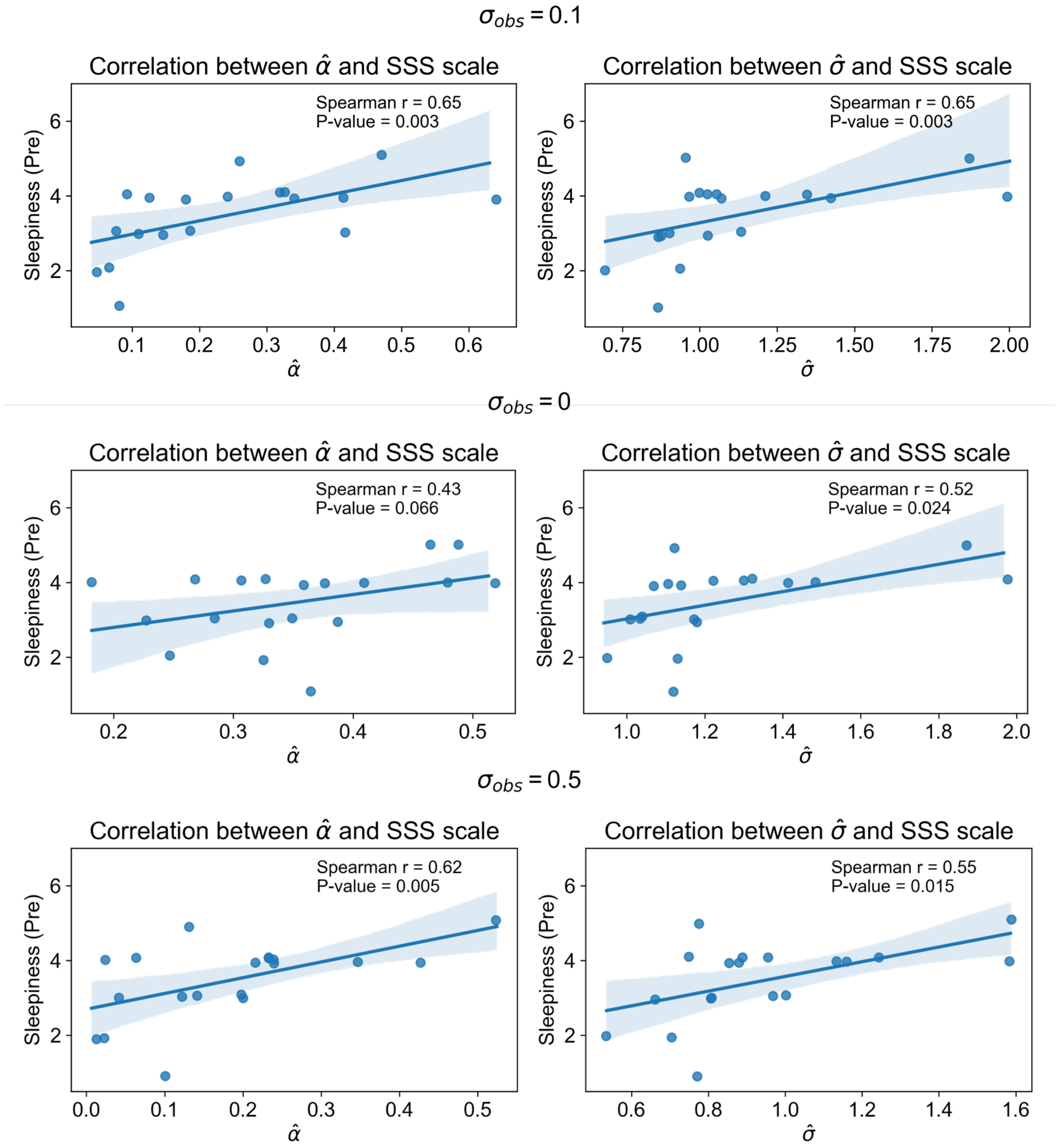

Fig C: Parameter–behavior associations remain consistent across σ_{obs} settings

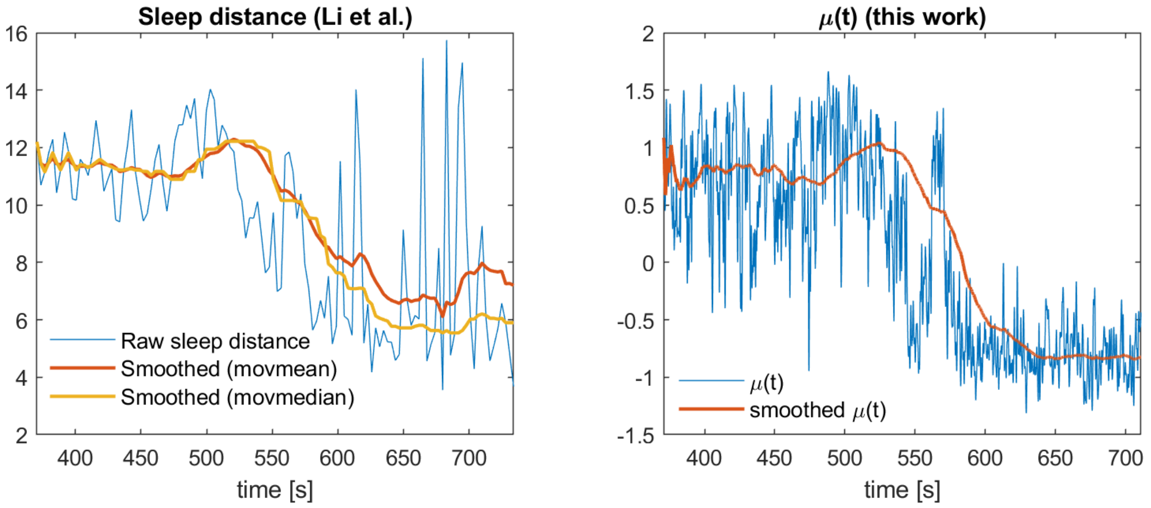

Fig D: Comparison of embeddings with Li et al. (2025) Left: The sleep distance (Li. et al) is computed as the Euclidean distance between the 49-dimensional feature vector for each 6-s window, and the sleep-onset centroid. The feature set includes power spectrum features, nonlinear features (catch22) and complexity features (e.g., Lempel–Ziv complexity). Before modeling it, Li et al. smooth the time-series with a 1-min moving-median filter (orange). Left: Our embedding, $\mu(t)$, is obtained by projecting the spectrogram bin to the dominant mode difference ($U_w^1 - U_s^1$). When smoothed using the same settings as Li et al. (red), the two curves present the same monotonic morphology.

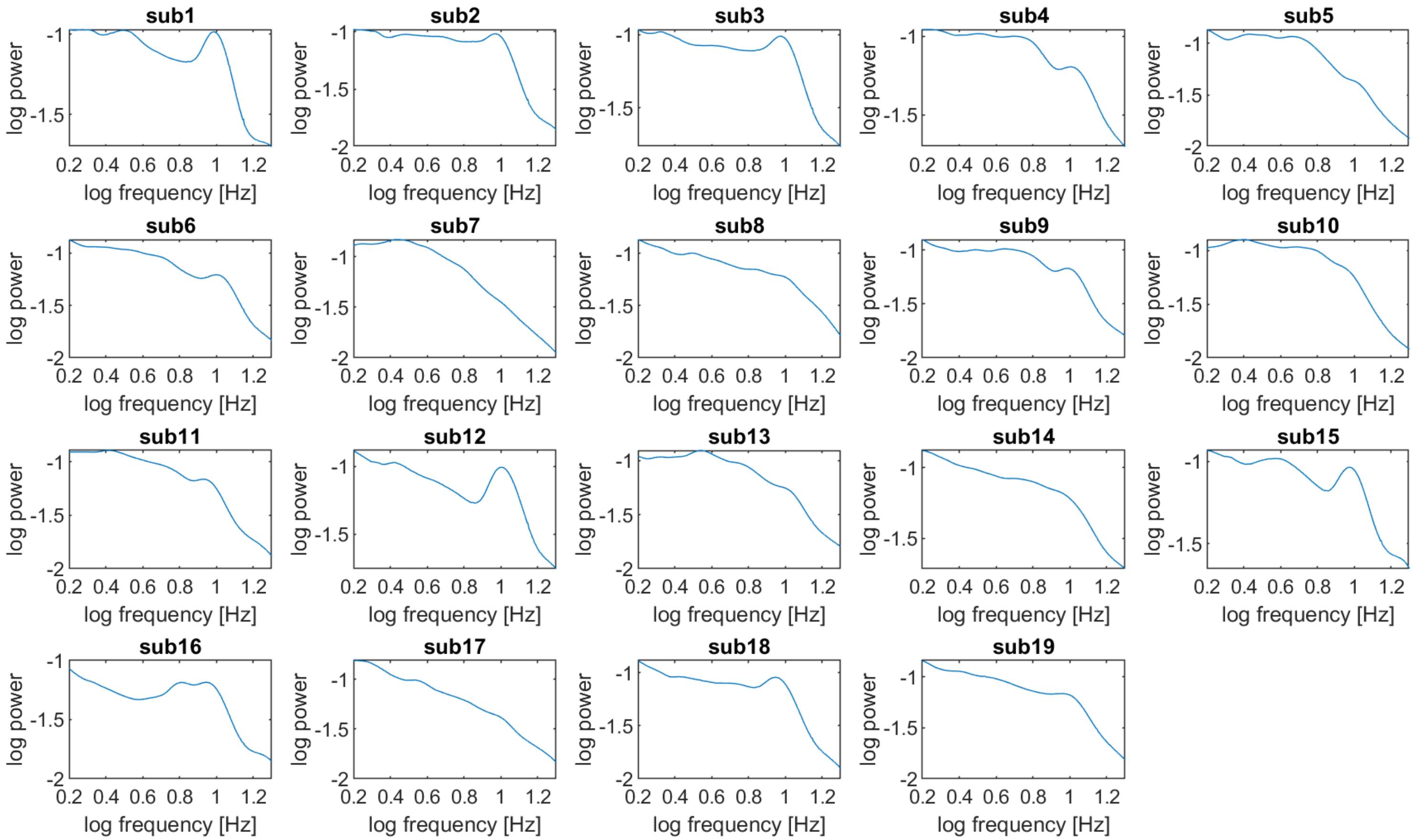

Fig E: First singular modes (U^1) of a global SVD analysis of the full SOP spectrogram, for all participants in our test dataset. The mode captures the broadband, power-law-like spectral structure which [?] normalizes out of their representation.

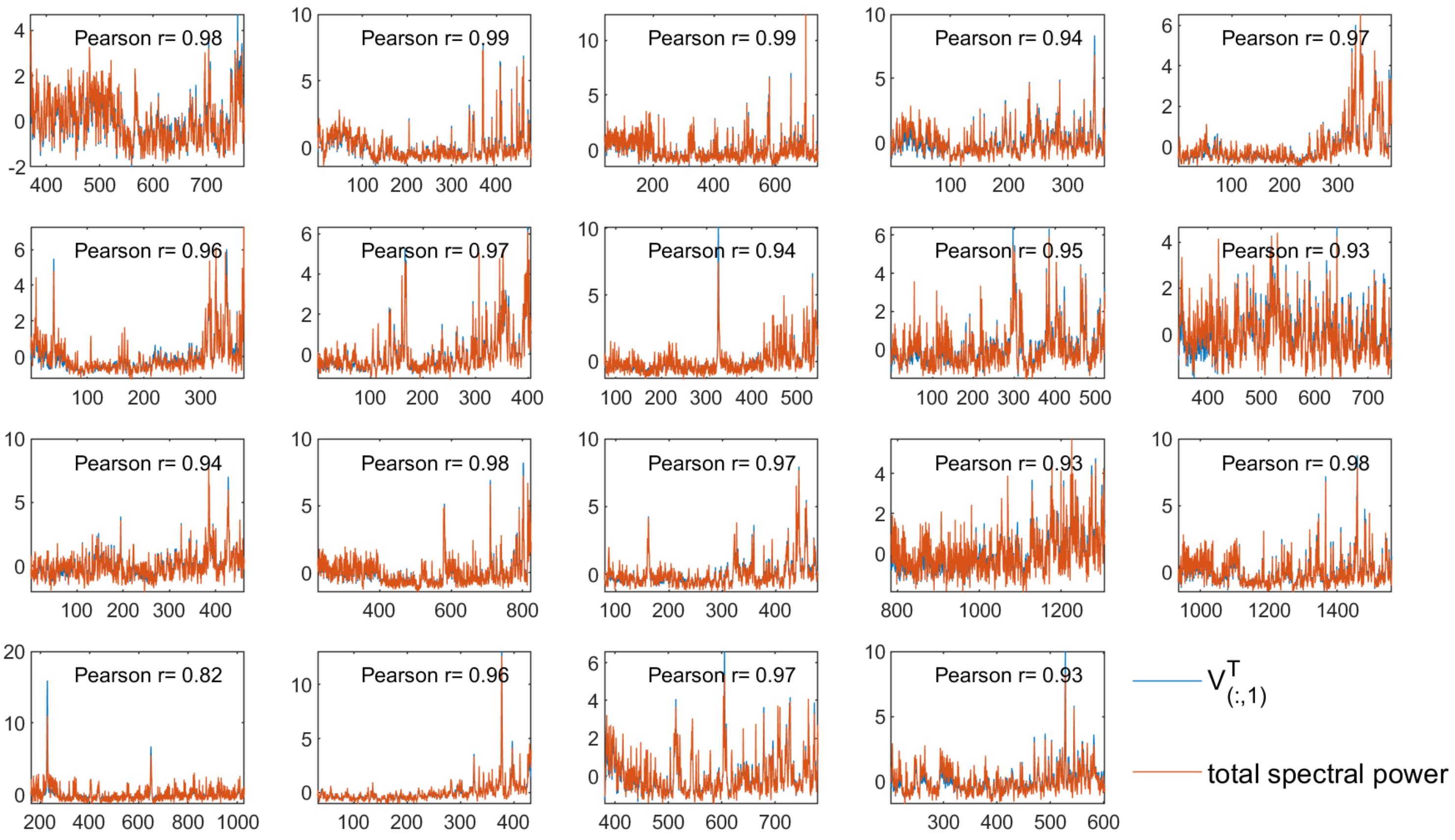

Fig F: Temporal weights of the first singular modes ($V^T_{(:,1)}$), superimposed to total spectral power

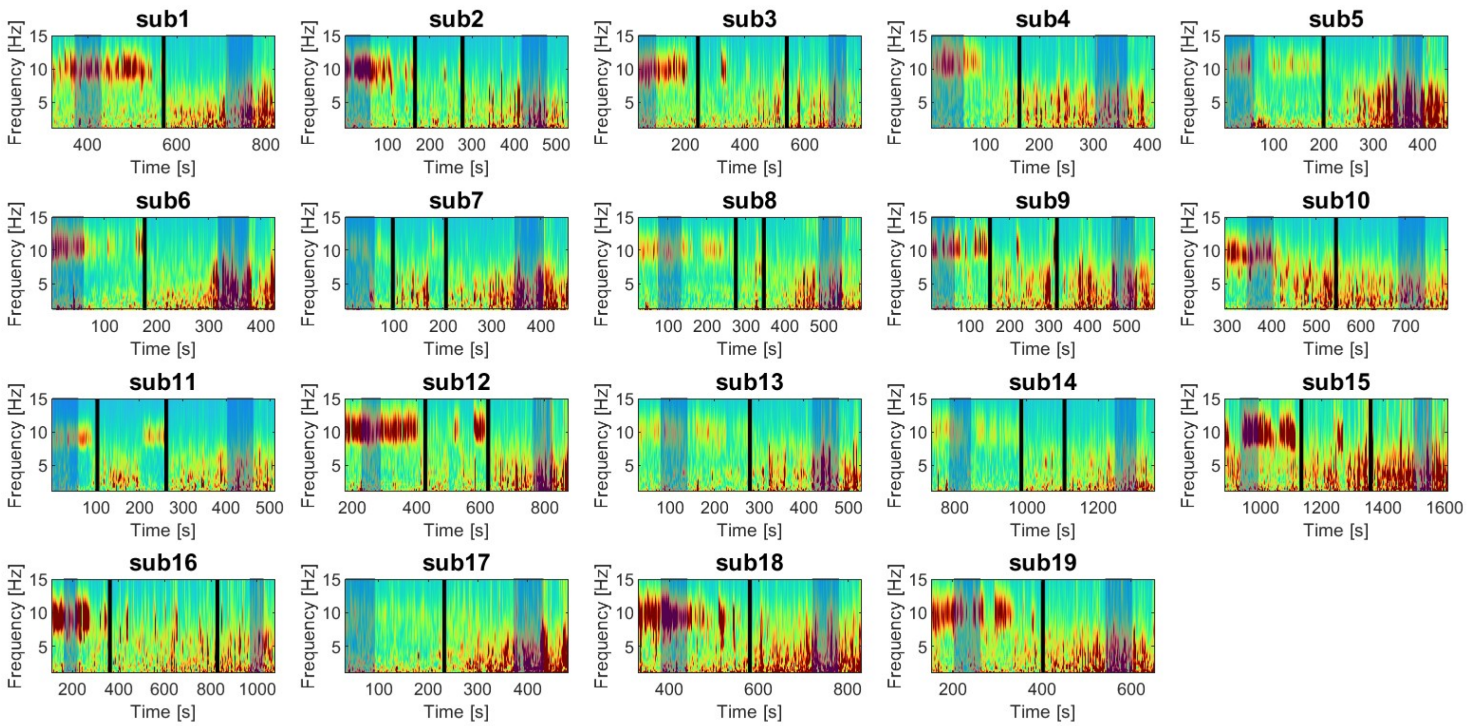

Fig G: Spectrogram localization of SOP windows for all participants. EEG wavelet spectrogram for the 19 participants. Each panel corresponds to one subject (**sub1–sub19**); the y-axis spans $0-15$ Hz and the x-axis shows time. The two *shaded* regions mark the first one minute ("wake"), and the last one minute ("sleep"), which are used as modes for the embedding. The central, unshaded block is the analysis window. Black vertical bars mark the (possibly identical) time points t_{start}, t_{end} used to define the analysis window: $[t_{start}$ - 200s., t_{end} + 200s.]. Across all individuals, the heuristic ratio $\text{amp}_\delta/\text{amp}_\alpha$ aligns well with the qualitative drop in α-power and rise in θ & δ-power, confirming the robustness of the SOP definition

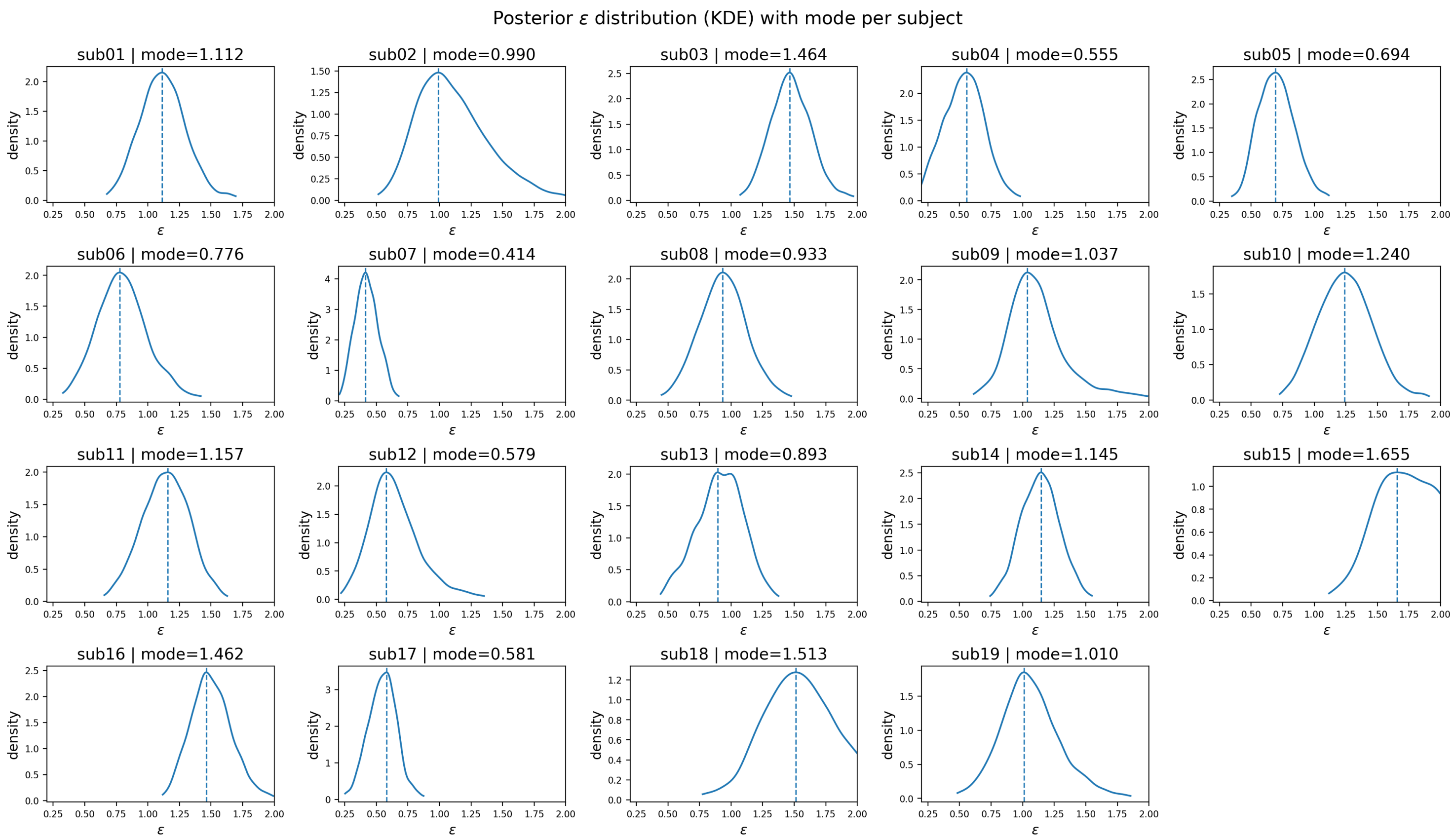

Fig H: Posterior distributions of ϵ. The model-fitting procedure estimates a time-scale adaptation factor ϵ, so the latent state dynamics can align with the unknown true time-scale of the experimental data. Figure here shows the posterior distribution of fitted time-scale factor ϵ for each experimental participant

.

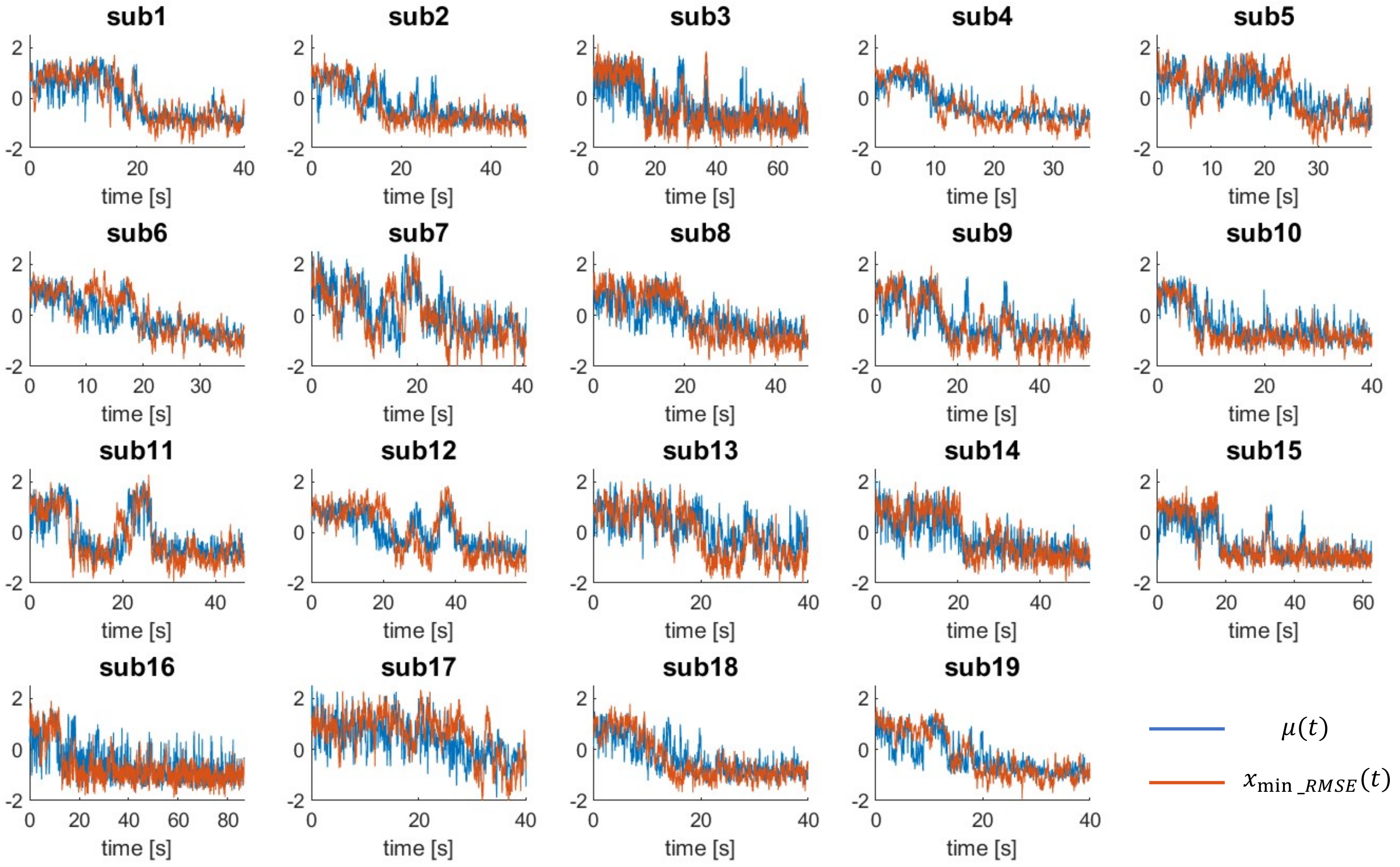

Fig I: Example posterior draws with minimal RMSE. For each subject we ran a MCMC inference over the model parameters and generated 4000 posterior predictive trajectories $x_{\text{sim}}(t)$ from the joint-posterior distribution. The orange line in every panel shows the simulated trajectory with the smallest root-mean-square error (RMSE) to the embedding $\mu(t)$ (blue)

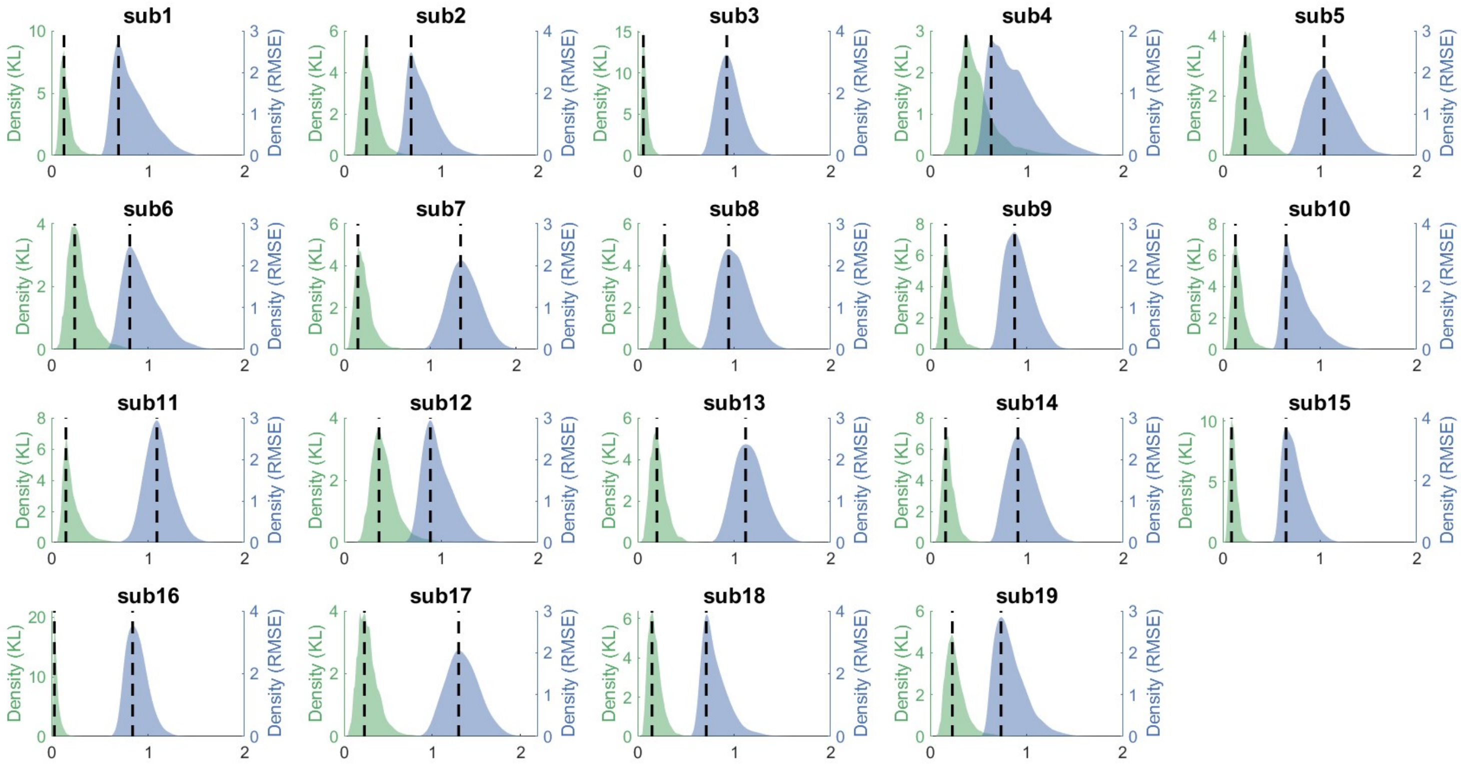

Fig J: Distribution of KL-divergence and RMSE over posterior samples. Posterior predictive checks were performed by forward simulation of 4000 trajectories from joint posterior distribution per participant and computing two complementary metrics: Kullback–Leibler (KL) divergence between the $\mu(t)$ and simulated trajectories (green, *left* axes) and point-wise RMSE (blue,*right* axes). Dashed vertical lines indicate the median of each distribution. For all subjects the bulk of the KL values lie below 0.16 and RMSE clusters around 0.8, confirming that a large portion of parameter draws generates trajectories statistically close to the data. Together with the illustrative trajectories in Fig **??**, these metrics demonstrate the overall adequacy of the inferred model.
