# Supplementary material for: Learning the bistable cortical dynamics of the sleep-onset period
Source: PLoS Comput Biol. 2026 Apr 30;22(4):e1014246. doi: 10.1371/journal.pcbi.1014246 (PMC13152216; doi:10.1371/journal.pcbi.1014246)
Supplement: Attachment — Submitted filename: change_log.pdf [file pcbi.1014246.s002.pdf]

Response to Reviewers / Change Log

Learning the bistable cortical dynamics of the sleep-onset period

Preprint: bioRxiv: 10.1101/2025.07.17.665340v3
Journal: PLOS Computational Biology
Date: August 22, 2025

Point-by-point change log (vs. previous bioRxiv version)

1. **Supporint Information** — The Whole supplementary materials were moved out of the main manuscript as an independent file *(Location: .)*

2. **Acknowledgements** — The funding sources are removed, which will go in the Financial Disclosure Statement *(Location: .)*
