# Supplementary material for: Learning the bistable cortical dynamics of the sleep-onset period
Source: PLoS Comput Biol. 2026 Apr 30;22(4):e1014246. doi: 10.1371/journal.pcbi.1014246 (PMC13152216; doi:10.1371/journal.pcbi.1014246)
Supplement: Attachment — Submitted filename: Response to reviewers.pdf [file pcbi.1014246.s003.pdf]

Response to Reviewers

Journal: PLOS Computational Biology

Manuscript title: *[Learning the bistable cortical dynamics of the sleep-onset period]*

Manuscript ID: [PCOMPBIOL-D-25-01784]

Corresponding author: [Zhenxing Hu]

Date: [16/02/2026]

Revision: [R1]

Response to Academic Editor

Dear Editor,

We thank you and the two reviewers for your detailed and positive assessment of our manuscript *"Learning the bistable cortical dynamics of the sleep-onset period"*, which we submitted for consideration in *PLOS Computational Biology*.

The two reviewers have provided a number of requests for clarification and additional analyses, which we detail below. As you will see, our responses to these comments are substantial. Among important changes made to the manuscript, we have clarified the link with our proposed embedding and the rank-2 structure of the EEG spectrogram (**Reviewer 1 - Comment 5** and **Reviewer 2 - Comment 3**); derived a new computable form for the model's per-timepoint likelihood, allowing to test model goodness of fit (**Reviewer 1 - Comment 8**); provided a detailed analysis of parameter degeneracy in the inference procedure, and confirmed that it didn't affect our results (**Reviewer 1 - Comment 9**); provided a reanalysis of the recent Li et al. Nature Neuroscience paper, clarifying the difference with our own work (**Reviewer 2 - Comment 1**); compared our proposed MCMC parameter inference procedure with an alternative method (Simulation-Based Inference; **Reviewer 2 - Comment 5**); and provided an additional analysis of how model parameters affect the number of switches between states (**Reviewer 2 - Comment 6**).

These new results, incorporated where appropriate in the manuscript and its now-numerous Supplementary Materials, make, in our opinion, for a much improved and clarified paper, which we are happy to resubmit for your consideration.

Kind regards,

Zhenxing Hu.

Response to Reviewer #1

General comment

In this work, Chen and colleagues proposes a dynamical systems model of sleep onset and a corresponding parameter inference procedure to extract per-individual sleep parameters from EEG data. The model is based on stochastic double-well dynamics with a monotonic sleep drive that shifts the potential function. The model parameters are fit to a 1D embedding of EEG spectral

mode differences between wake and sleep. The authors validate the inference procedure on simulations, and apply it on real EEG data. The inference procedure is generally accurate, produces faithful reconstructions of the spectrogram, and results in parameter estimates that are correlated with subjective reports of sleepiness.

In general, the model seems to me well-motivated, simple, and elegant. The double-well dynamics is a natural choice for such a twostate transition between wake and sleep, and inferring the model parameters based on a low-dimensional decomposition of EEG spectrogram appears reasonable as well. The MCMC-based inference procedure works reasonably well, though where it's less accurate may be important to consider (see comment below). The application on real EEG recordings and the resulting association with sleepiness are interesting and suggest future potential in linking dynamics at the phenomenological level to underlying physiology though the authors are careful in not speculating specific neural substrates. Overall, the paper was clearly structured, written, and nicely illustrated, especially the schematic of the workflow in Figure 1.

Response. We thank the reviewer for their positive assessment of our work.

Comment 1

The 2-state dynamic makes sense considering wake to sleep, but what about the other sleep stages? They have distinct spectral signatures, while some states (like REM) could be considered to be in between wake and sleep. Related, one would intuitively think that such a model, in reverse, could be used to explain sleep to wake transitions, though I don't think such a symmetry is present?

R1. An interesting question. While our model was specifically designed to study the SOP, a transition which the community explicitly acknowledges exhibits complex temporal dynamics [Lacaux et al., 2024], our embedding and modeling strategy can be applied to any phenomena where two regimes each have a dominant spectral component (approximately rank-1 SVD structure) and these dominant spectral modes (U_w^1 and U_s^1) are sufficiently different. In principle, if other stable sleep states fit that criteria, it would therefore be possible to also model these transitions. Whether such modeling would reveal interesting patterns of noise-driven bistability, on the other hand, is an open question. As the reviewer suggests, one possible candidate for such modeling is the symmetric transition from sleep to wake, or sleep *offset*. The first minutes following awakening from sleep are typically marked by reduced vigilance, increased sleepiness and impaired performance, a state referred to as sleep inertia [Trotti, 2017]. While this transient phenomena is comparable in duration with the sleep onset period (M=15.9min in [Kim et al., 2026]) and appears to be associated with specific EEG spectral signatures (e.g., increased EEG delta power and delta/beta ratio at 5-min post-awakening, compared to pre-nap wakefulness and 25-min post-awakening; [Vallat et al., 2019]), most research so far has taken a point-estimate approach (e.g. comparing immediately after awakening, and 30min-1hr later) and paid little attention to its dynamical nature [Occhionero et al., 2021]. Another potential application of the model is the direct transition from wake to REM that is sometimes seen in narcolepsy patients [Weinhold et al., 2011].

Changes. We have added a short discussion in the revised manuscript in the **Line 338-340** of the discussion section to discuss this possibility.

Comment 2

Another point worth discussing are sensory disturbances in the process of falling asleep, including e.g., self-motion.

R2. Agreed. While our primary intention was to model undisturbed transitions to sleep, the model could provide a scaffold to model how external inputs may interact with the intrinsic SOP dynamics. It is indeed well-known that many people use e.g. musical stimuli to ease their transition to sleep [Buus et al.,]; conversely, there are also well-studied adversarial effects of e.g. environmental noise on sleep quality [Basner and McGuire, 2018]. The reviewer is right that such perturbations (positive or negative) could also be internally generated, such as self-motion or snoring [Hoffstein et al., 1995]. If one has access to direct measurements of the perturbation signals (e.g. real-time acoustic recording, or stimulation), one could possibly incorporate these as additive control signals in a $\dot{X} = AX + BU$ manner. In the long-term, such an extension could potentially open the way for closed-loop control on the model, e.g. synthesizing a sound control signal able to interact with the wake-to-sleep transition dynamics, in a way reminiscent of other closed-loop stimulation strategies using in later stages of sleep [Ngo et al., 2013].

Changes. We have added a short discussion of these extra possibilities in **Line 341-343** of the revised manuscript.

Comment 3

The spectrogram representation of the EEG ignores phase, in particular coupling across frequencies, which could be an informative marker (e.g., up-down states). Are any such considerations relevant in the context of this model? Or is the goal simply to look for maximum SNR to separate the two stages?

R3. In an abstract way, our model simulates bistable transitions between two states, which are represented by SVD modes in a possible large feature space. Here, we use the EEG spectrogram magnitude as input to that SVD, but the reviewer is correct that the model could be extended to include more, or alternative, feature representations of the EEG, essentially modifying the definition of the brain states that we are studying.

In a recent paper, Li et al. [Li et al., 2025] use a set of 50 EEG features to construct an embedding similar to ours (see additional discussion of that paper in response to Reviewer 2 below), and conduct a PCA analysis of what groups of features express what aspect of the SOP dynamics. Their dataset includes a number of features that loosely encode phase information (e.g. theta and alpha band coherence, phase-amplitude coupling), and they find that those in particular do not help representing the system's state (but, intriguingly, they suggest that they correlate with the bifurcation control signal - what we describe as $\beta(t)$ here). Based on these results, we would thus be tempted to say that phase information may not add much to our specific research question here, but that of course could be the topic of future work.

Changes. We have added a few sentences in the paper's discussion (**see Line 329-331**) stating that the same modeling strategy could be extended to other feature sets.

Comment 4

What motivates the form of the transition as a tanh, as opposed to, e.g., rectified linear, or an asymmetric function?

R4. We don't have a strong justification here. In biophysically grounded models of the wake-sleep cycle [Yang et al., 2016], the sleep drive (often decomposed into homeostatic $H(t)$ and circadian $C(t)$ components) acts as a slow, continuous modulator of the underlying switch dynamics. Over the relatively short SOP windows, we do not aim to model the full physiology of $H(t)$ and $C(t)$ but instead use a local phenomenological approximation that captures the qualitative features observed in practice: an approximately stable pre-onset epoch, a finite transition window, and a post-onset saturation. A hyperbolic tangent function provides the simplest smooth, bounded parameterization of this period of interest, with two interpretable parameters (the start (t_0) and the rate (α) of the sleep-drive ramping). We did not adopt a rectified-linear (ReLU) ramp because it is either non-saturating (unless a cap is added) or becomes piecewise with explicit change-points, and we did not introduce asymmetric saturating forms because they add flexibility that is not required for our aims here and would complicate interpretation.

Changes. We have added a paragraph (**see Line 529-543**) to the manuscript to motivate the choice of the hyperbolic tangent form $\beta(t)$ for the drive.

Comment 5

Related, is there a way to retrieve / infer the wake and sleep modes in an unsupervised fashion, e.g., without manually labeling the wake and sleep periods? Another way to conceptualize it is to fit the spectral mode (difference), along with the transition and noise parameters, directly and jointly from the spectrogram data? The discussion mentions the second SVD component of the overall spectrogram, but in the context of a forced energy transfer between the top sleep and wake modes.

R5. Thank you for raising this point. To clarify the first part of your comment, first, we do not use the manual sleep scoring to label the wake and sleep periods to fit the model. Rather, we specify a fixed, subject-independent *criteria* for data-driven state change detection (based on the δ to α power ratio) as described in the manuscript, and define the SOP window relative to these automatically detected boundaries.

That said, the reviewer is correct that, we do the embedding and the modeling *sequentially*, independently from one another. That brings up the more interesting question of jointly inferring (i) an intrinsic coordinate/mode and (ii) a parsimonious governing equation directly from high-dimensional observations. Recent work in the group of e.g. Nathan Kutz [Champion et al., 2019, Gao et al., 2025] explicitly emphasizes that model discovery depends on finding a coordinate system in which the latent dynamics admit a simple representation; they address this by learning the coordinate transform (encoder/decoder) while enforcing sparse latent dynamics via SINDy.

The first thing we can report on that is that, actually, our sequential procedure for identifying the begin and end state in the SOP is empirically equivalent to applying SVD on the whole SOP period. As seen in Fig. 1 , a whole-spectrogram SVD gives two main components, one of which correlates with total spectrogram power and the latter, with $\mu(t)$ as we define it 'manually'. This suggests that our proposed embedding is relatively agnostic and data-driven, as it coincides naturally from the space of maximum variance in the data.

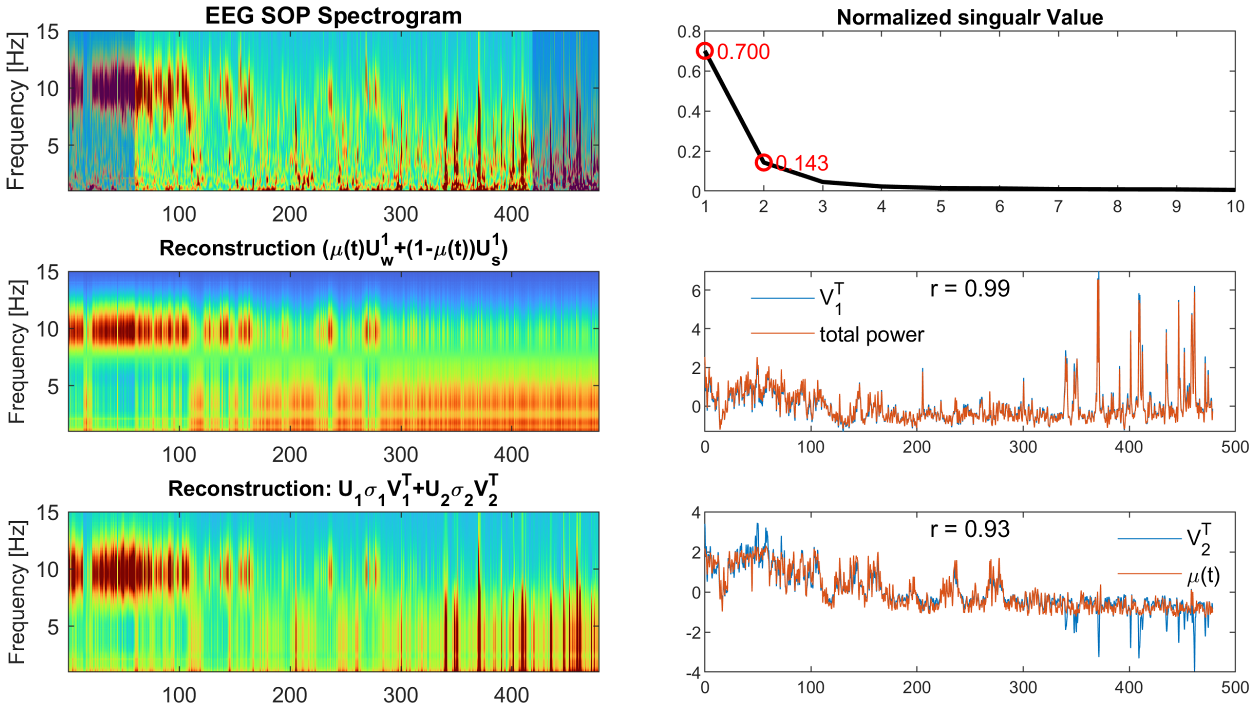

Figure 1: **Relating between 1-D transition coordinate $\mu(t)$ to a Rank-2 SVD decomposition of the full SOP spectrogram (single-subject example).**
Left column: Top, the wavelet-based representation of the SOP spectrogram. Middle, reconstruction using 1-D embedding ($\hat{S}(f,t) = \mu(t)U_w^1(f) + (1-\mu(t))U_s^1(f)$). Bottom, a rank-2 SVD reconstruction of the full SOP spectrogram ($\hat{S}(f,t) = U_1\sigma_1V_1^T + U_2\sigma_2V_2^T$). *Right column:* Top, explained variances of the normalized singular values from the SVD on the full SOP spectrogram. Middle, the first right-singular vectors V_1^T closely tracks total spectral power (sum across frequencies) ($r = 0.99$). Bottom, the second right-singular vectors V_2^T closely tracks the transition coordinate $\mu(t)$ ($r = 0.93$).

Secondly, to more directly address the reviewer's comment, we have experimented with SINDy-SHRED [Gao et al., 2025], a recent architecture that uses GRU (Gate Recurrent Unit) to map a lagged history of sparse sensor measurements into a low-dimensional latent state with a shallow recurrent decoder network to reconstruct the high-dimensional observation. The model is **jointly** trained with a SINDy regularization term that encourages the latent state to follow a sparse ODE from a prescribed function library. Our initial experiments show that, if we restrict the latent dimension to be 1, and limit the SINDy library to the 2nd/3rd-order polynomials, the resulting embedding is qualitatively very similar to $\mu(t)$ we constructed (Fig. 2). However, because SINDy-SHRED is primarily designed for regimes in which the data are well-approximated by a dominant low-dimensional autonomous/stationary ODE with only mild stochasticity, the recovered latent dynamics associated to that embedding typically reduce to slowly decaying components, failing to model the noise-driven transitions or hidden/non-autonomous drive that are the focus of our interest here. Jointly recovering both the embedding and noise/transition parameters would probably require a stochastic/non-stationary driven identification framework beyond the scope of SINDy-SHRED, or the present paper.

In sum, while our proposed embedding $\mu(t)$ is indeed designed manually, it is closely consistent both with data-driven alternatives from the whole-spectrogram SVD or SINDy-SHRED. While jointly inferring the governing model together with transition/noise parameters from spectrogram data is interesting from a data-deriven modeling perspective, these results suggest, in our view, that our current proposal captures a representative share of the phenomenon.

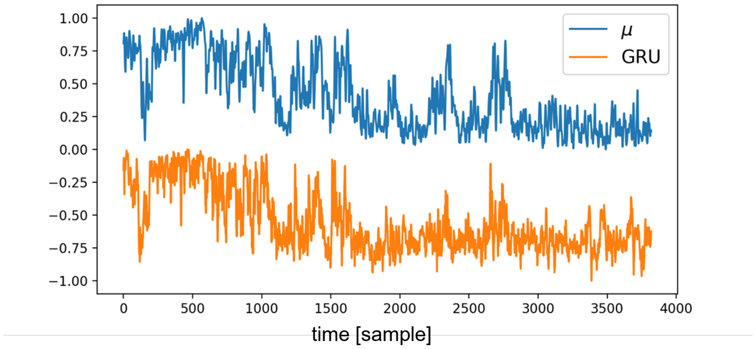

Figure 2: **GRU/SINDy-SHRED as a data-driven alternative for constructing a 1-D embedding.** Time courses of our SVD-based transition coordinate $\mu(t)$ (blue) and the 1-D latent state inferred by SINDy-SHRED using a GRU encoder (orange) from the same SOP spectrogram. The two embeddings show qualitatively similar wake-to-sleep progression.

Changes. Because the SINDy-SHRED data is only anecdotal, we have not included it in the revised manuscript. We have put the SVD decomposition result in the manuscript's discussion (Figure 12), and have mentioned that possible future work could extend it to variables (e.g. V_1^T/total power) which are not part of the model for now (**see Line 427 - 444**).

Comment 6

Time in the non-stationary simulations (e.g., Fig 6 a and d) is on the order of ˜50 seconds, but in the real data the transition appears to happen in about 10$\times$ longer timescale (400–500 s), and with variable absolute duration (N). Are the conclusions from the simulated scenarios still valid given this discrepancy, especially since the inference procedure tends to overestimate alpha at lower values (corresponding to longer timescale transition), or is it simply a matter of rescaling? It would be informative to see what the non-stationary simulations in the "realistic" timescale look like, as well as the fitted posterior predictive samples.

R6. Sorry for the confusion. Illustrations for simulated data in the manuscript is not in second units, but in units of the model integration step. Second, while the duration of EEG spectrograms are indeed of the order of 500s, we downsample the resulting embedding by a factor of 10 (**line 404** in the original manuscript) before fitting the parameter. Therefore, the effective physical timescale represented by the model is stretched by x10 relative to the original spectrogram grid. Under this mapping, a transition that spans ~ 50 model steps corresponds to a transition on the order of ~ 500s in the original recording, consistent with the reviewer's estimate for the real data.

In addition, it's important to highlight that we also add a provision for time-scale fine-tuning, in the form of an unknown factor $t^* = \epsilon t$ which is fitted as part of the model, so that the latent state

can align with the unknown true time-scale of experimental data. We show here the posterior distribution of that timescale parameter $t^* = \epsilon t$, across subjects (Fig. 3). The posterior modes cluster around 1 ($\bar{\epsilon} = 1.011,\ \mathrm{SD}(\epsilon) = 0.36$), indicating that the inferred transition timescale in model units matches our non-stationary simulation setting.

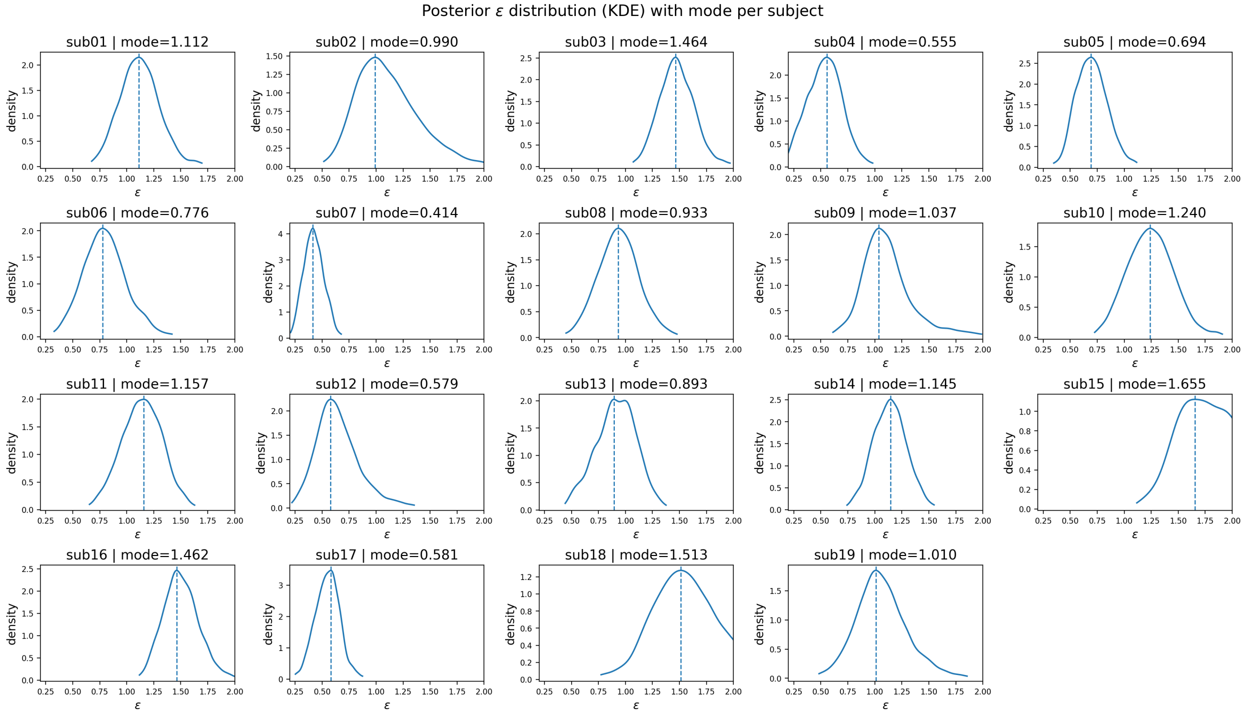

Figure 3: **Posterior distributions of ϵ:** The model-fitting procedure estimates a time-scale adaptation factor ϵ, so the latent state dynamics can align with the unknown true time-scale of the experimental data. Figure here shows the posterior distribution of fitted time-scale factor ϵ for each experimental participant

.

Changes. We have clarified in Figure caption where the time axis is in units of model integration and added details about the timescale estimation, including Fig. 3 here, in supplementary material (**SI Figure 8**).

Comment 7

Is KL computed on distributions collapsed over time (e.g., Fig 7b)?

R7. Yes. Because we only have one value per time point in the data, we cannot form a full empirical distribution at each time and then compute a KL at every time point. Instead, in Fig. 7, the KL divergence is computed between the empirical distribution of the observed trajectory and the marginal distribution obtained from all posterior simulations, both collapsed over time (Fig. 7b). This provides a global measure of how similar the overall amplitude distribution of the simulated trajectories is to that of the empirical embedding.

Changes. We have clarified this procedure in the main text (**Line 596**), as well as in the Figure caption.

Comment 8

In addition to KL and RMSE, because the trajectories have an additive stochastic component in sigma assumed to be a Gaussian, one could in theory compute the per-timepoint data likelihood under the fitted models. What do some of the worse / non-minimal RMSE/KL samples look like?

R8. The reviewer is correct that the time-series of likelihood can be computed from successive state estimates under the fitted models, and that this likelihood can be informative of how the MCMC procedure manages the compromise between true model noise (σ) and observation noise (σ_{obs}), and more generally, goodness of fit. Concretely, the Eq. 1 defines the one-step predictive density of the next observation obs_{t+1} given the current sampled latent state x_t and parameters θ obtained by marginalizing over the unobserved next state x_{t+1} across posterior draws.

$$p(\mu_{t+1} \mid x_t, \theta) = \int \underbrace{p(\mu_{t+1} \mid x_{t+1}, \sigma_{obs})}_{\text{observation model}} \underbrace{p(x_{t+1} \mid x_t, \sigma, \alpha, t_0, \epsilon)}_{\text{transition model}} \, dx_{t+1}. \tag{1}$$

For a given draw s (parameters $\theta^{(s)}$, latent state $(x_t^{(s)}, x_{t+1}^{(s)})$), the complete one-step joint density can be decomposed into one observation term and one transition term (Eq. 2) by taking the logs.

$$\log p\Big(\mu_{t+1}, x_{t+1} \mid x_t, \theta^{(s)}\Big) = \log p\Big(\mu_{t+1} \mid x_{t+1}, \sigma_{\text{obs}}^{(s)}\Big) + \log p\Big(x_{t+1} \mid x_t, \alpha^{(s)}, t_0^{(s)}, \sigma^{(s)}, \epsilon^{(s)}\Big). \tag{2}$$

The observation model in Eq. 3 is a Gaussian distribution with mean equal to the latent state and variance $(\sigma_{obs}^{(s)})^2$

$$p\Big(\mu_{t+1} \mid x_{t+1}, \sigma_{\text{obs}}^{(s)}\Big) = \frac{1}{\sqrt{2\pi}\,\sigma_{\text{obs}}^{(s)}} \exp\left(-\frac{(\mu_{t+1} - x_{t+1})^2}{2\left(\sigma_{\text{obs}}^{(s)}\right)^2}\right). \tag{3}$$

The transition density in Eq. 4 comes from Euler-Maruyama discretization of the cubic SDE. For one given draw s, the one-step transition is conditionally Gaussian with mean given by the drift updates and variance set by the diffusion term:

$$p\Big(x_{t+1} \mid x_t, \alpha^{(s)}, t_0^{(s)}, \sigma^{(s)}, \epsilon^{(s)}\Big) = \frac{1}{\sqrt{2\pi\left(\sigma^{(s)}\right)^2 \epsilon^{(s)} \Delta t}} \exp\left(-\frac{\left[x_{t+1} - x_t - \epsilon^{(s)} f(x_t, t; \alpha^{(s)}, t_0^{(s)}, \epsilon^{(s)})\Delta t\right]^2}{2\left(\sigma^{(s)}\right)^2 \epsilon^{(s)} \Delta t}\right). \tag{4}$$

These two likelihood terms typically trade off: if σ_{obs} is set at a larger value, the fitting procedure allows the state x_t to deviate more from the observation obs_t, and to better fit cubic dynamics; setting σ_{obs} at a smaller value constrains the state to fit the observation, at the possible expense of the transition likelihood. This trade-off is illustrated in Figure 4 for the case of Subject 01.

This trade-off doesn't appear to be consequential on the reported correlation with subjective sleepiness, which is to say that the particular association of cubic parameters α and σ seen among our test participants is not critically driven by changes of σ_{obs}. Figure 5 shows the regression plot of sleepiness on α and σ for $\sigma_{obs} \in [0, 0.1, 0.5]$, with no strong effect on the observed trend.

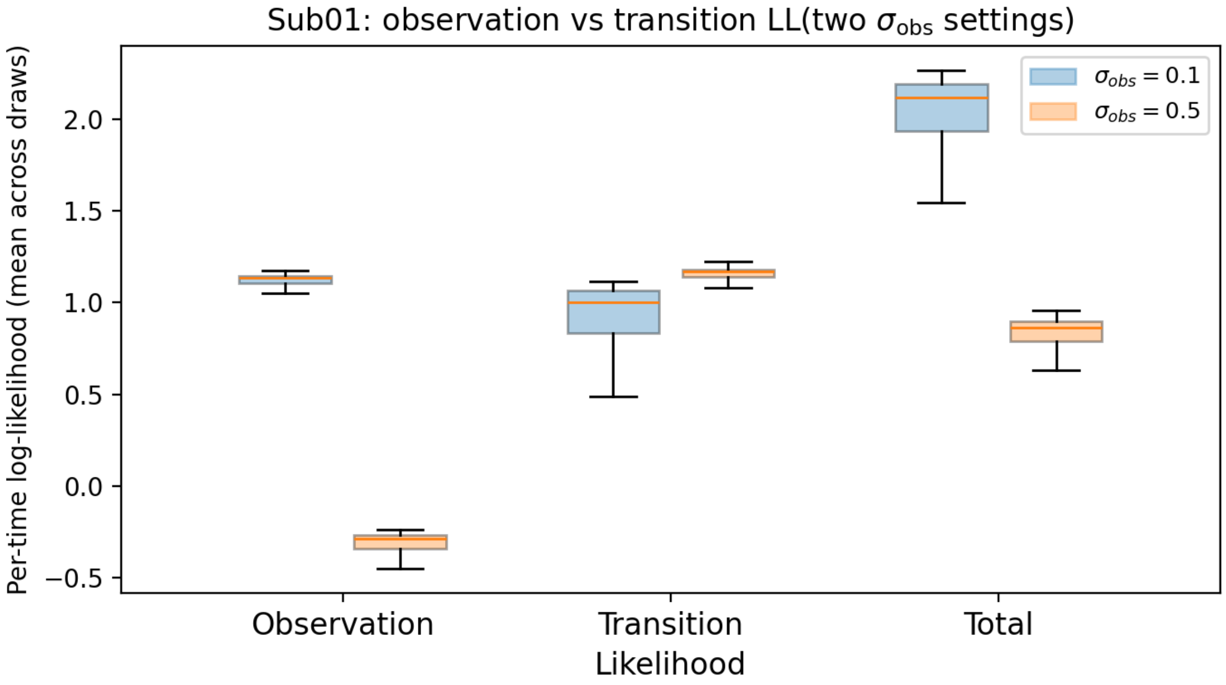

Figure 4: **Observation–transition trade-off induced by the observation noise level.** This illustrative example shows how changing the observation noise σ_{obs} change the two additive components of the one-step log-likelihood during MCMC sampling: the observation term $log\, p(obs_t \mid x_t, \sigma_{obs})$ and the transition term $log\, p(x_t \mid x_{t-1}, \alpha, \sigma, \epsilon)$ as well as their sum ("Total"). We compute a per-time point log-likelihood by taking the mean value over the posterior draws. Each sample in the box corresponds to one time time point.

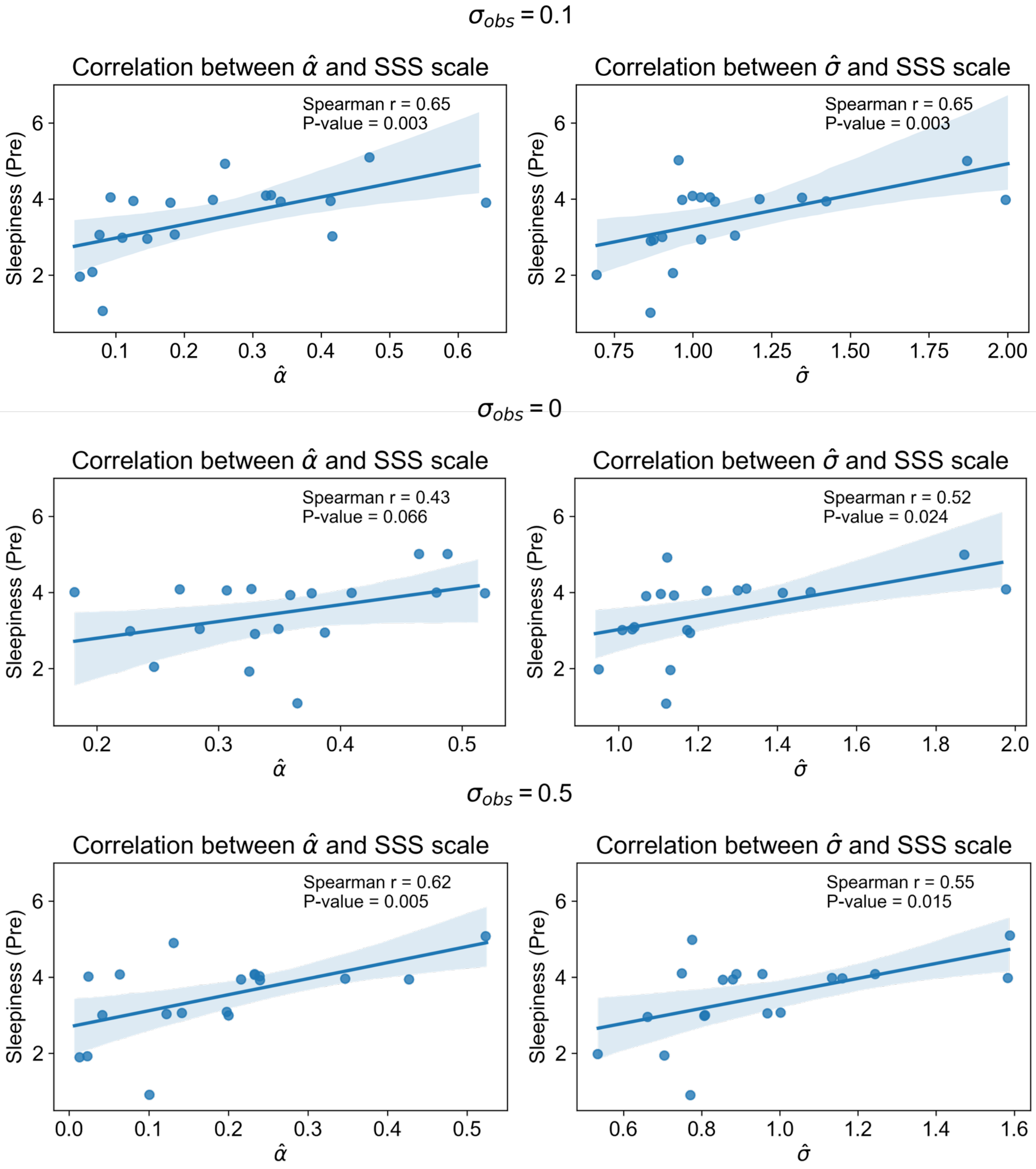

Figure 5: **Parameter–behavior associations remain consistent across σ_{obs} settings**

The ratio of the per-timepoint observation and cubic transition likelihood is informative of how the MCMC procedures attributes events in the experimental data to either σ-driven transitions in the cubic dynamics (i.e. amplitude increments on $\dot{x}_t$), or to σ_{obs}-driven observation noise (i.e. amplitude increments on x_t) that operate outside of the state dynamics. Figure 7 shows the time series of both (z-scored) observation-noise and transition likelihood, as well as the z-scored difference of observation - transition likelihood, along with the modeled trajectory, for each of the 19 participants in our test dataset. While the comparison of these time-series is necessarily a bit anecdotal, it appears to confirm the ability of MCMC to trade off noise and model fit: in subject 01 for instance (Figure 6, high noise in the initial part of the embedding is attributed to observation noise, possibly because low noise in the final part has lead to model to adopt a relatively small σ_{cubic} (comparison A); large transitions of comparable amplitudes may be attributed to observation noise or to the dynamics (comparison B) depending on e.g. their temporal position along the sigmoidal change of landscape.

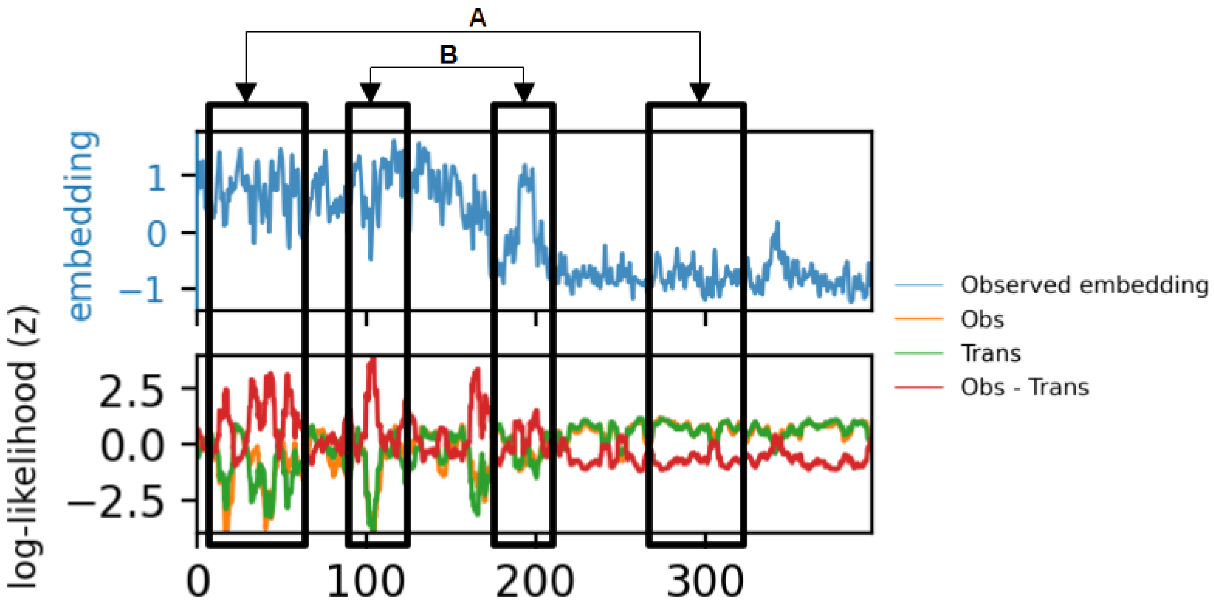

Figure 6: **Annotated detail from Figure 7: Subject 01**

This suggests that the average/mode value of the distribution of transition likelihood is potentially indicative of the model's goodness of fit for individual participants, i.e. how well the participant's embedding correspond to our assumption of stochastic bistable dynamics. Figure 8 shows the distribution of per-timepoint likelihood, as well as its mode value, for each of the 19 participants in our test datasets. Two participants (Sub17: mode_tr=0.62 and Sub13: mode_tr=0.59) appear to have relatively low likelihood ($<$10%-percentile), and these two participants correspond to embeddings which indeed do not entirely fit our expected phenomenology (slow, monotonous drift with no evident bistable jumps, refer e.g. to Figure 7).

As a confirmatory analysis, we have verified that the reported correlation between cubic parameters and subjective sleepiness was not driven by these 2 poorly-fitted participants: it is not the case (Figure 9).

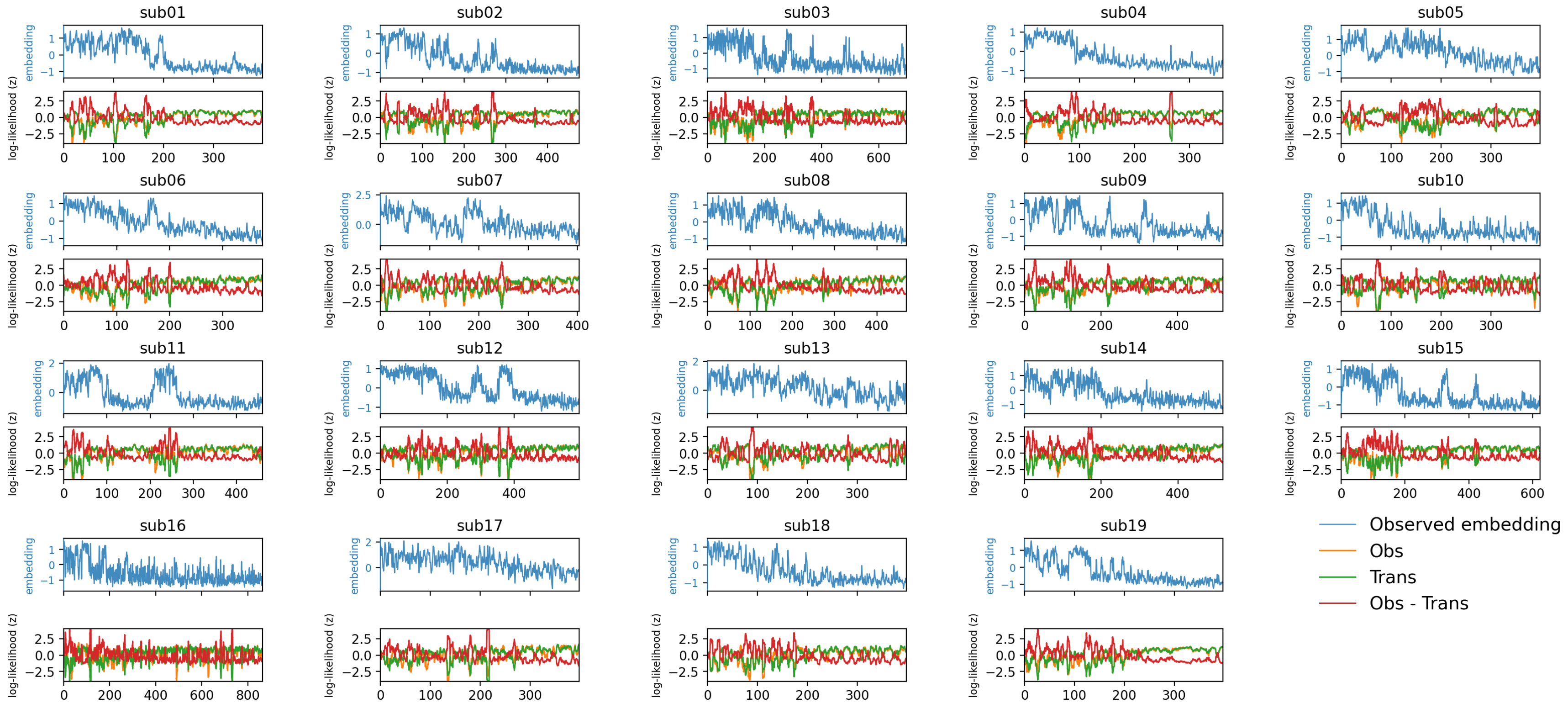

Figure 7: **The ratio of the per-timepoint observation and cubic transition likelihood is informative of how the MCMC procedures attributes events in the experimental data to either cubic dynamics or observation noise.** Time series of z-scored observation-noise (orange), transition likelihood (green), as well as the z-scored difference of observation - transition likelihood (red), along with the modeled embedding (blue), for each of the 19 participants in our test dataset.

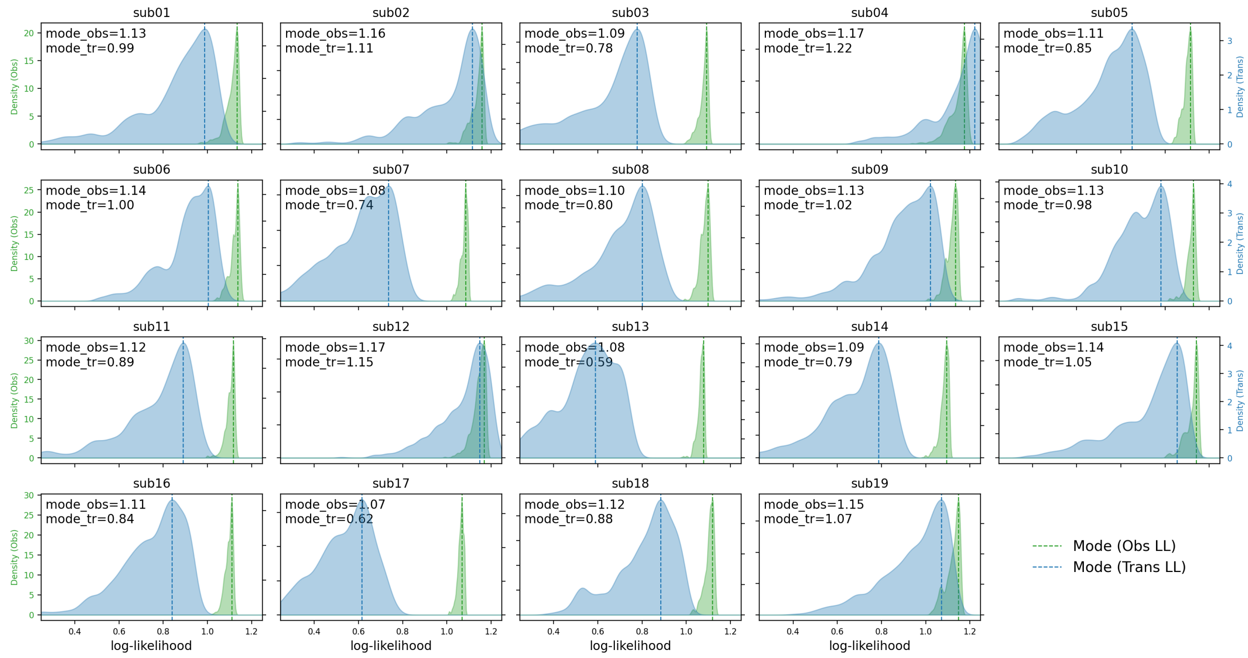

Figure 8: **KDE of per-time observation-noise and transition log-likelihood.** For each subject, we firstly compute the per-timepoint observation-noise and transition log-likelihood for all 4000 posterior draws, and then take the median across draws at each time point, yielding a single time series transition log-likelihood values. We then aggregate these values across time and plot their kernel density estimate (KDE). The dashed vertical line marks the mode of the KDEs.

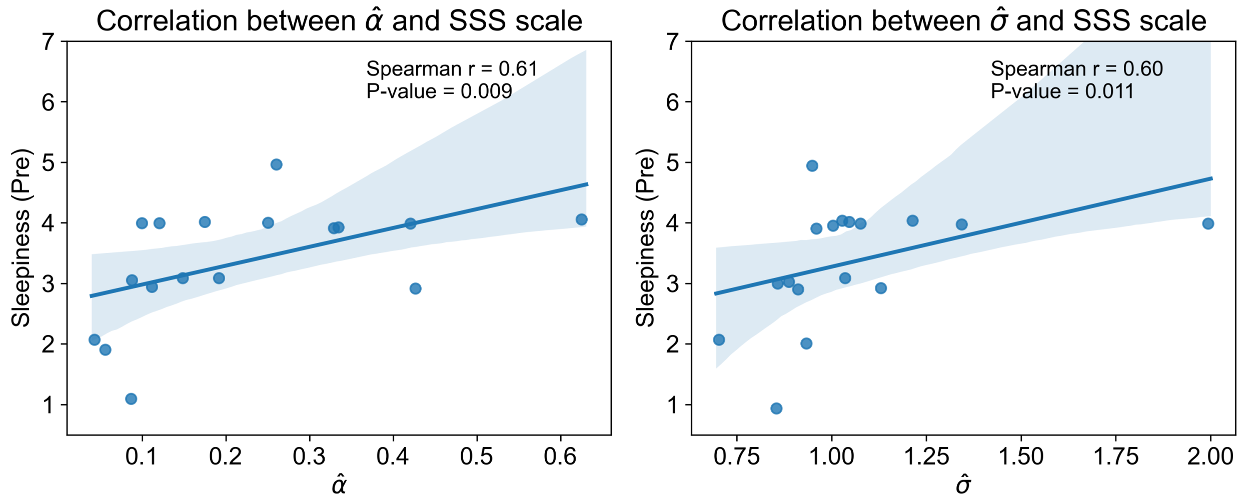

Figure 9: **The reported association between cubic parameters and subjective sleepiness is not driven by participants who have low model fit.** Regression plots excluding the 10%-percentile of mode of transition likelihood (subjects 13 and 17)

Changes. We have added paragraphs in the model evaluation (**Line 598 - 616**) and results (**Line 242 - 254**) section of the paper mentioning an alternative analysis with log-likelihood, and incorporated Figure 8 into the revised manuscript. We have added Figures 7 and 6 as Supplementary **SI Text 1**. We have also reported alternative correlation results in the results section, and added the corresponding figure 9 and Fig. 5 in Supplemental Material, as **SI Fig. 2 and 3** resp.

Comment 9

In Figure 8, estimated alpha (transition slope) and sigma (noise level) are both found to correlate with pre-nap sleepiness, with potential proposed interpretations for both. It's intuitive that the speed of transition into sleep should correlate with sleepiness, but the correlation with noise amplitude is interesting and surprising. One potential explanation that's not necessarily "neural" is that the inference of these two parameters jointly results in a correlation between the two, i.e., there is degeneracy between the two parameters given an observation. Is this typically observed, e.g., in inferred parameters from simulated data? Concretely, looking at the joint distribution of inferred values in Fig 6 for example, are there systematic correlations between the values (and errors) of the different parameters.

R9. This is an important question. There are two points that need to be disentangled: first, it is possible that the participants' estimated modes for alpha and sigma are correlated, and that this may 'explain away' one of the two correlation results we report for subjective sleepiness. Second, it is also possible that the MCMC fitting procedure creates degeneracy between alpha and sigma given an observation, i.e. within a single participant's posterior distribution. While it is possible that the latter (strong, within-participant degeneracy) results in the former (e.g. in the situation where all within-participant ridges are aligned in the same principal direction between participants), we do not think that it is necessarily the case, as we will show here.

First, the reviewer is right that there is a positive association between alpha and sigma in our experimental dataset (Fig. 10), which is statistical with an OLS regression: $\sigma \sim \alpha$: $\beta = 1.135$, $t(17) = 2.74$, $p = .014$. In statistical terms, this collinearity makes it difficult to separate the contribution of both parameters to subjective sleepiness SS. While both parameters are statistically predictive of sleepiness when tested individually ($SS \sim \alpha$: $\beta = 3.59$, $t(17) = 2.91$, $p = .01$; $SS \sim \sigma$: $\beta = 1.64$, $t(17) = 2.65$, $p = .017$), neither of them are in a joint OLS model ($SS \sim \alpha + \sigma$; α: $\beta = 2.48$, $t(16) = 1.72$, $p = .11$; σ: $\beta = 0.971$, $t(16) = 1.38$, $p = .18$), which translates the model's incapacity of conclusively attributing variance to one or the other predictor. Both model comparisons ($SS \sim \alpha$: $AIC = 49.9$ *vs* $SS \sim \sigma$: $AIC = 51.0$) and residual tests ($SS \sim residual(\alpha \sim \sigma)$: $\beta = 2.48$, $t(17) = 1.45$, $p = .16$ *vs* $SS \sim residual(\sigma \sim \alpha)$: $\beta = 0.97$, $t(17) = 1.14$, $p = .27$) indicate weak evidence that α may be more predictive that σ (which matches the reviewer's intuition), but we do not think that evidence is conclusive.

Second, in the MCMC estimation procedure, we indeed find some (weak, r=0.2) degree of degeneracy between α and σ in the joint posterior distribution for a given fitted trajectory. Upon analysis, the relation between α and σ is mediated by the time-scale correction parameter ϵ (see **Comment 6**, above): in the model, α and ϵ appear as a product in $\beta(t) = tanh(\alpha\epsilon(t - t_0)$ and $f(x, \beta(t))\epsilon$, while σ is multiplied to ϵ through the diffusion term ($\sigma\sqrt{\epsilon}$). This coupling leads to degeneracy, as keeping $\sigma\sqrt{\epsilon}$ approximately constant yields a strong anti-correlation between σ and ϵ, and keeping $\alpha\epsilon$ constant also yields a $\alpha - \epsilon$ tradeoff.

To quantify these relations in simulated data, we performed an additional simulation in which we

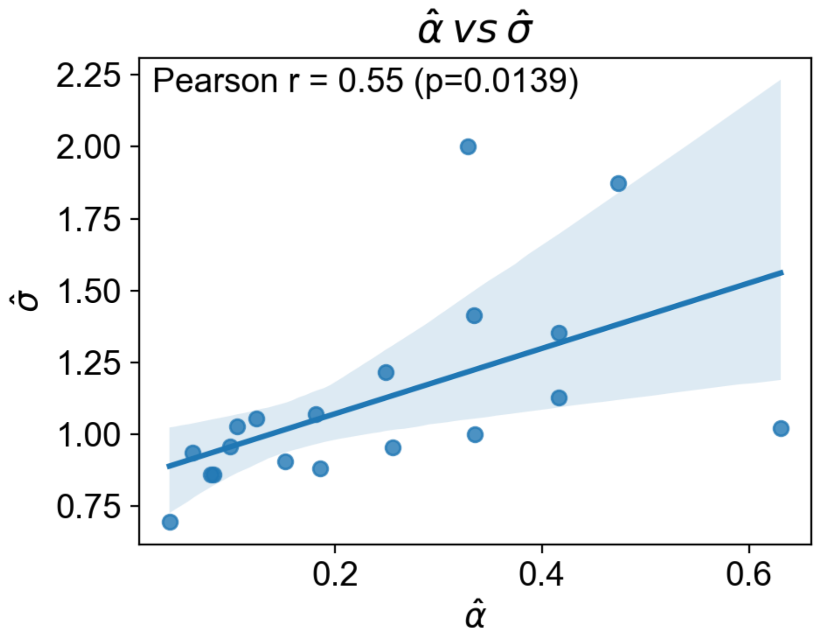

Figure 10: **Across-subject co-variation between $\hat{\alpha}$ and $\hat{\sigma}$.** Each point corresponds to one subject's posterior point estimates (e.g., marginal mode ($\hat{\alpha}$,$\hat{\sigma}$), with the fitted regression line and Pearson correlation reported in the panel.

deliberately introduced a time-scale mismatch. Specifically, we simulated trajectories using the SDE integration step ($\Delta t_{sim} = 0.01$), formed observations by downsampling every 2 steps (yielding $\Delta t_{obs} = 0.02$), and fit the downsampled observations using the original model, allowing him to compensate mismatch between Δt_{model} and Δt_{obs} with ϵ. While the posterior modes of ϵ all appropriately converged to a factor of 2 (M=1.90), we observed systematic within-trajectory posterior correlations among parameters (Fig .11-left), confirming the intuitions from model structure above. The relation was weakly negative between α and ϵ (the greater the time-scale factor, the steeper the slope), strongly negative between σ and ϵ (the greater the time-scale factor, the lower the noise) and, as a result, weakly positive between α and σ. Identical relations were found, again within-posterior, when fitting the real experimental data (Fig .11-right).

Importantly though, parameter degeneracy in the joint posterior distribution within-trajectory does not automatically induce correlation of these parameters across trajectories. In the time-mismatch simulation, although each run shows a positive correlation between α and σ within its posterior, the across-trajectory correlation of point estimates (for a grid of 7 α values and 4 σ values) remains near zero ($r = 0.05, p - value = 0.32$). More generally, these posterior trade-offs do not imply that parameters cannot be accurately recovered. Under the simulated time-scale mismatch, $\hat{\epsilon}$ converges to the predicted value (2) with a small bias, σ is accurately recovered, and α is recovered in a monotonic (rank-preserving) manner across ground-truth values (Figure 12; while this figure resembles the parameter estimation figure in the manuscript, the latter was done by forcing $\Delta t_{obs} = \Delta t_{sim}$). The fact that the between-individual $\alpha \sim \sigma$ is seen in the experimental dataset, but not in simulated data, therefore indicates that this co-variation is, for a good share, a separate phenomenon from parameter degeneracy in the within-trajectory posterior geometry.

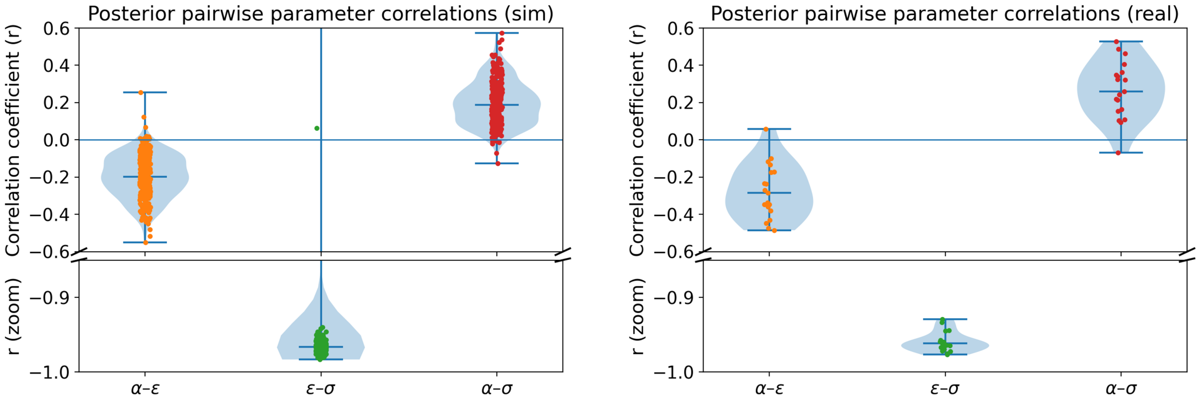

Figure 11: **Within-trajectory posterior parameter correlations in both simulation and real data.** For the time-scale–mismatch simulation (left), we computed pairwise Pearson correlations between posterior samples of $(\alpha, \epsilon, \sigma)$ within each MCMC run given one observed trajectory. We repeated this for 10 simulated trajectories per parameter setting on a grid of 7 α values and 4 σ values (total $7 \times 4 \times 10$ trajectories, t_0 is fixed as 50. The same within-subject posterior-correlation analysis is shown for the real-data fits across participants (right))

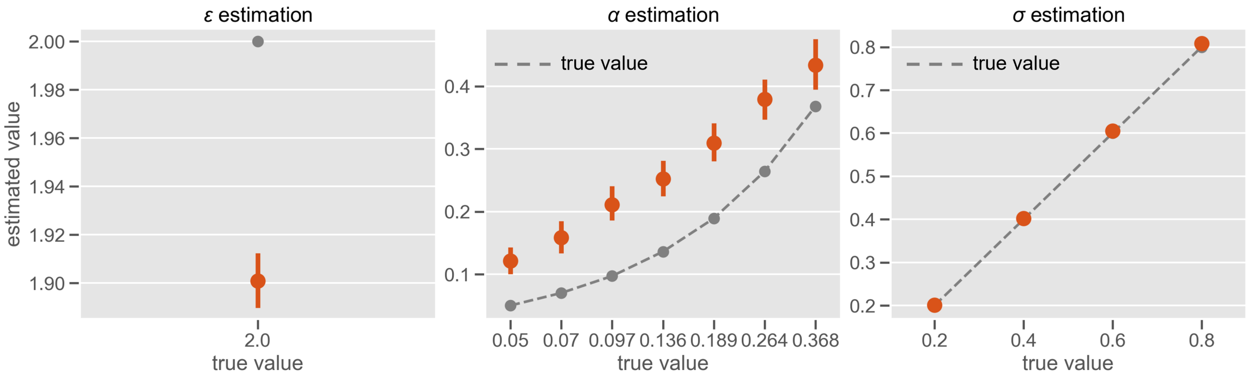

Figure 12: **Parameter recovery remains accurate under imposed time-scale mismatch despite posterior degeneracy.** We simulated trajectories on a grid of 7 α values and 4 σ values (with t_0 fixed), generating 10 trajectories per parameter setting. Trajectories were downsampled to create a time-scale mismatch and then fit with the original model while allowing ϵ to be inferred. Points show posterior point estimates with confidence interval across trajectories for each true parameter value. The gray dashed line indicates the identity trend (true value), highlighting that ϵ and σ are recovered well and $\alpha$is recovered monotonically (rank-preserving).

In summary, the reviewer is right to point that the experimental data shows a moderate correlation between α and σ and that, at the same time, posterior degeneracy exists between these 2 parameters. However, our additional experiments shows that this degeneracy is an expected structural feature of the model parameterization, linked to time-scale adaptation, and that it does not prevent reliable recovery of the underlying parameters under time-scale mismatch scenarios, providing no evidence that the co-variation between α and σ isn't caused by genuine inter-individual differences.

Changes. We have updated the manuscript to clarify that, because of the across-individual correlation of α and σ in the experimental dataset, we have no evidence that both α and σ independently predict subjective sleepiness, and that future work should clarify these links (**see Line 282 - 298**).

Comment 10

I'm not sure I understood what is meant by "how possibly" / phenomenon models(line 44–46). Based on the explanation that follows, that means non-mechanistic / non-neural, or specifically that their parameters were not estimated per-individual? I'm asking this in the context of clarifying the contribution here, and whether the advancement is in a new model, a parameter estimation procedure, or both.

R10. We agree with the reviewer that, for our purpose, this classification of 'how-possibly' vs 'how-actually' models adds more confusion than clarification. In fact, our original introduction even possibly misreads the distinction brought in by Craver [Craver, 2007]. In Craver's words, our model *"describes how a set of parts and activities [a sigmoid, a cubic landscape] might be organized such that they exhibit the explanandum phenomenon [but we] have have no idea if the conjectured parts exist"* - something Craver actually describes as "how-possibly".

In any case, what we meant to emphasize in the introduction is the link with real EEG data (embedding) and the possibility of doing parameter estimation at the individual level. This key difference with the literature held at the time of submission, but a recent paper [Li et al., 2025] also brings in the same elements, albeit a slightly different modeling goal. We have therefore rewritten the introduction to take into account this updated state of art, and let go of the how-possibly/actually distinction. In short, given the updated state of art, our contribution is both a new model (SOTA models that fit data are deterministic, and we model stochasticity) and a parameter estimation procedure (SOP models that incorporate stochasticity do not have parameter estimation).

Reviewer 2 - Comment 1 below gives a detailed discussion of the similarities and differences between our work and [Li et al., 2025]. In terms of contribution (the reviewer's question), if there is any biological position that our paper entails, it is to look at neural noise as a mechanism for the sleep onset period. [Li et al., 2025] makes the assumption that noise and bistable jumps can be smoothed out, and are therefore not mechanistically important for the transition to sleep; similarly, classical biophysical models for the transition to sleep are also heavily focused on landscape change, i.e. the deterministic drive of the phenomenon. We on the contrary are making noise one of the two crucial components of our model, to the fact that, strictly speaking within our modeling framework, systems with $\sigma = 0$ will never transition to sleep. Here, we show that the addition of noise can make the model more phenomenally adequate than a purely deterministic model (in line with recent calls to model the "complexity" of the SOP, [Lacaux et al., 2024]), and we show that the estimated noise correlates with subjective sleepiness. While we are not trying to speculate where that source of stochasticity comes from (but see Robinson's work on narcolepsy linking frequent bistable transitions between states with reduced activity of orexinergic neurons of the lateral hypothalamus [Fulcher et al., 2014a]), we at least suggest it should be a target for future research.

Changes. We have rewritten the paper's introduction to take into account the updated state of art [Li et al., 2025], and clarified our methodological and conceptual contribution (**see Line 42 - 54**).

Comment 11

Relatedly, one stated contribution of the model and associated inference workflow is that the inferred model parameters may shed light on the mechanisms of sleep onset, as shown by its correlation with sleepiness report. In other words, they may serve as model-based biomarkers for sleep stages and disorders. While this is true, are there simpler metrics one can compute from the data itself, without the complex MCMC inference procedure, that correlates well (or better) with the subjective ratings? Alternatively, could one potentially make physiological interpretations of the parameters, which would provide additional mechanistic and treatment value beyond data-driven metrics (though it is explicitly discussed in the limitations that b(t) is not necessarily physiologically grounded). If not, how should one view the current model beyond a "reanalysis" of the EEG data? In general, while the model is intuitive and elegant, I think the stated contribution section (lines 44–54) is a bit narrow in scope.

R11. We appreciate this opportunity to clarify our goal and contribution. Our primary goal is phenomenological explanation. We aim to provide an interpretable dynamical description of the sleep-onset period (SOP) at a fine temporal resolution, including its intermittent switches, bistable behavior and inter-individual variability. We are not positioning this work as an optimized biomarker pipeline for sleep-onset disorders, nor claiming that our inferred parameters are the best possible predictors of subjective ratings. Rather, we frame the contribution as a compact dynamical description of full progression of SOP and its inter-individual variability. In this context, we also explicitly position our work with respect to the recent [Li et al., 2025], which models the wake-to-sleep transition using a saddle-node bifurcation perspective (see also **Comment 10** above, and **Reviewer 2, Comment 1**). The key point is that we provide a complementary view on the SOP dynamics as we target different phenomena/time-scales. The work of [Li et al., 2025] focuses on the final "no-return" tipping point and applies heavy temporal smoothing, which naturally favors a deterministic low-dimensional description of the SOP as a slowly-varying, near-monotone trajectory (see Fig. 16 for a comparison of the pre-processing pipeline of [Li et al., 2025] and ours, on the same data). In contrast, we target the full SOP progression without smoothing away fast-scale dynamics, making intermittent bistable excursions prominent. This motivates a stochastic account and explicit parameterization of individual variability.

So could simpler and direct metrics also correlate with sleepiness? Yes, but we contend that they would often be more ambiguous. We agree that one can compute similar summaries directly from the derived embedding without model inversion. For example, one could do a sigmoid fit to $\mu(t)$ to characterize the slope and transition timing, or simply count the wake-sleep switches/reversals during SOP (as we do below by the way, see **Reviewer 2 - Comment 6**). The limitation is that such feature-based summaries can be mechanistically non-identifiable, in the sense that identical switch counts may arise from qualitatively different underlying dynamics. In our settings, a larger number of reversals can be produced either by a slowly evolving landscape with modest stochastic forcing, or by a more rapidly evolving landscape combined with larger noise. In other words, a simple summary can confound how fast the landscape evolves with the magnitude of stochastic forcing. Our estimation procedure is meant to separate these contributions by estimating the changing rate of the landscape (α) and stochastic forcing (σ). More broadly, our motivation is grounded in a general principle of biological dynamics in which observed dynamics often arise from the interaction between slowly-varying control parameter (here a time-varying landscape during SOP) and fast-scale fluctuations (here represented as stochastic forcing). In such systems, slow parameter drift continuously reshape stability while rapid fast-scale fluctuation drive intermittent excursions. Our position is that modeling the interaction between these time scales is essential for

capturing the phenomenology of SOP.

In this context, the role of model inversion (here via MCMC) is not just to "fit a curve" (or "re-analysing EEG") but to disentangle contributions that are conflated in direct feature summaries and to quantify uncertainty in that decomposition. This is aligned with a broader viewpoint in stochastic dynamical modeling of brain/clinical phenomena [Roberts et al., 2017]: model inversion is valuable precisely because it aims to estimate latent dynamical parameters and to separate biological signal from noise/measurement effects, rather than relying solely on surface-level summaries.

Changes. We have revised the contribution paragraph, expanded the discussion to make our scope and claims more precise, as well as updating our abstract (**see Line 42-52 in introduction section and Line 377-406 in the discussion section**).

Comment 12

There are some previous works on decomposing EEG spectrograms specifically via SVD / PCA from KJ (Kai) Miller that are probably worth discussing and relating to (around line 67).

R12. Thanks for this recommendation. In [Miller et al., 2009], the PCA basis (principal spectral components, PSCs) is learned from an ensemble of PSD snapshots (the ordering of epochs is ignored), and is then used to obtain time-resolved trajectories by projecting wavelet-based spectra onto the 1st PSC. A key practical difference is the experimental/phenomenological setting. Miller et al. study event-locked, repetitive motor behavior, where the mean spectral shape is treated as a stable baseline confounding trial-to-trial/task-related deviations. Hence, they center the spectrum relative to the log of the mean spectrum (i.e., subtracting the log of mean spectrum from the log of the original spectrum) to cancel out the exponent part ($f^{-\chi}$) of the power law phenomenon, ($P \sim Af^{-\chi}$), hence they intentionally focus on the background gain, A. In contrast, during SOP, the aperiodic spectral profile (including slope/tilt) can drift over time and is itself a central phenomenon we aim to model. Therefore, we do not apply a mean-spectrum ratio/centering step, which would suppress precisely the non-stationary spectral structure that is informative in our setting.

Miller's PCA framework is in fact most directly comparable to a global SVD analysis of the full SOP spectrogram. In [Miller et al., 2009], the 1st principal spectral component is primarily flat across all frequencies, while the classic peaked rhythms (α/β range) of motor tasks are accounted for by the 2nd and 3rd components. In our context, the first global SVD mode ($U_{(:,1)}$) would capture the broadband, power-law spectral structure (Fig. 13) and its weighting across time ($V^T_{(:,1)}$) is highly correlated with the temporal fluctuations of broadband spectrum power (Fig. 14). The 2nd global mode ($U_(\;:, 2)$) captures the changes of spectral shape that are associated with the wake-to-sleep transition, and closely matches our embedding computed by interpolating start and end-state SVDs (U^1_w and U^1_s, Fig. 15), as already discussed in **Comment 1** and Fig. 1.

Changes. We have added the KJ (Kai) Miller's work in the introduction (**see line 73 - 80**). We have included Fig. 15 as part of **SI Text 2**, and Fig. 13 and 14 in Supplementary Materials, as **SI Fig 5 and 6**, resp.

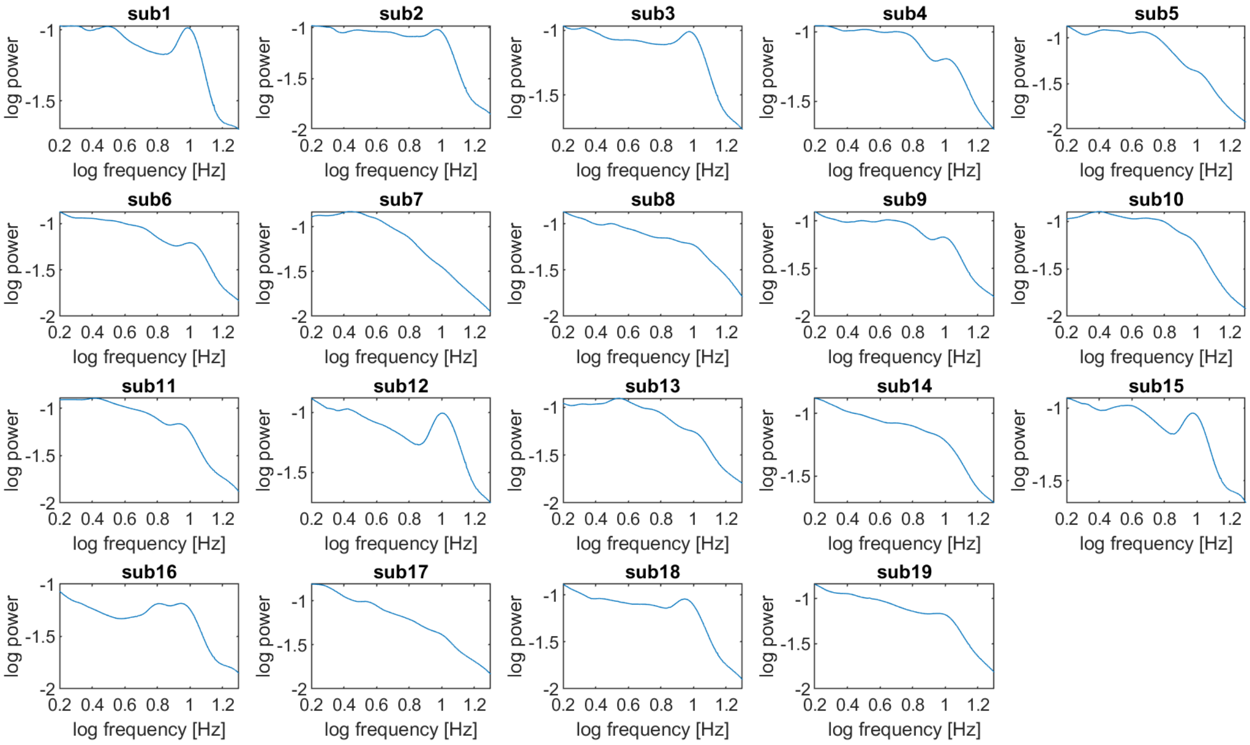

Figure 13: **First singular modes (U^1) of a global SVD analysis of the full SOP spectrogram, for all participants in our test dataset.** The mode captures the broadband, power-law-like spectral structure which [Miller et al., 2009] normalizes out of their representation.

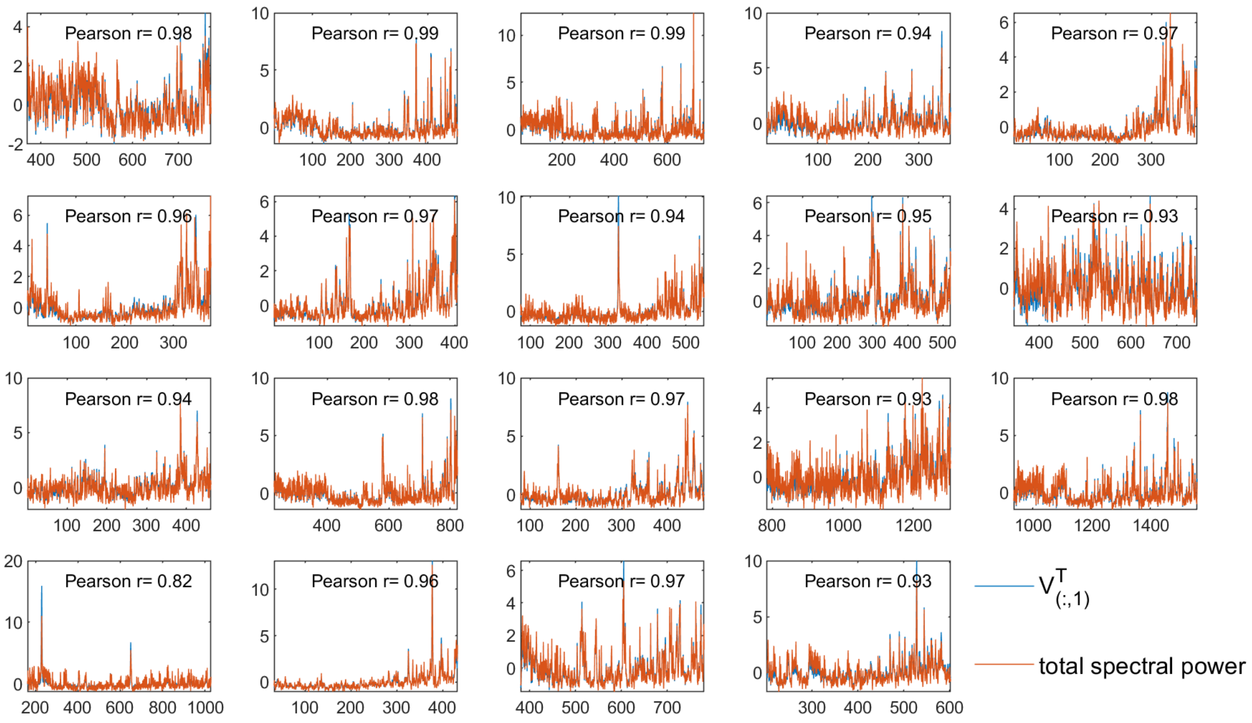

Figure 14: **Temporal weights of the first singular modes ($V^T_{(:,1)}$), superimposed to total spectral power**

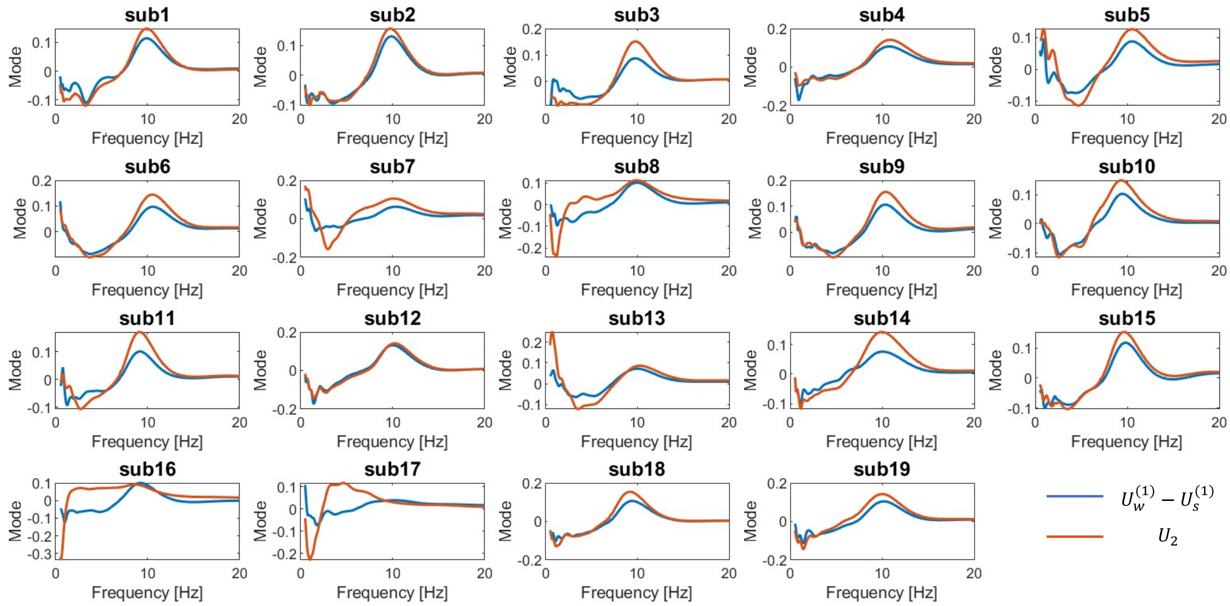

Figure 15: **Comparison between the 2nd global SVD mode $U_{(:,1)}$ and $U_w^1 - U_s^1$.**

References

Andrillon et al., 2021. Andrillon, T., Burns, A., Mackay, T., Windt, J., and Tsuchiya, N. (2021). Predicting lapses of attention with sleep-like slow waves. *Nature communications*, 12(1):3657.

Basner and McGuire, 2018. Basner, M. and McGuire, S. (2018). Who environmental noise guidelines for the european region: a systematic review on environmental noise and effects on sleep. *International journal of environmental research and public health*, 15(3):519.

Buus et al., . Buus, R. M., Genovese, S., and Jespersen, K. V. The art of sleep: examining sleep strategies in the general population with a focus on the use of music for sleep. *Journal of Sleep Research*, page e70006.

Champion et al., 2019. Champion, K., Lusch, B., Kutz, J. N., and Brunton, S. L. (2019). Data-driven discovery of coordinates and governing equations. *Proceedings of the National Academy of Sciences*, 116(45):22445–22451.

Craver, 2007. Craver, C. F. (2007). *Explaining the brain: Mechanisms and the mosaic unity of neuroscience*. Clarendon Press.

Deistler et al., 2025. Deistler, M., Boelts, J., Steinbach, P., Moss, G., Moreau, T., Gloeckler, M., Rodrigues, P. L., Linhart, J., Lappalainen, J. K., Miller, B. K., et al. (2025). Simulation-based inference: A practical guide. *arXiv preprint arXiv:2508.12939*.

Fulcher et al., 2014a. Fulcher, B. D., Phillips, A. J., Postnova, S., and Robinson, P. A. (2014a). A physiologically based model of orexinergic stabilization of sleep and wake. *PLoS One*, 9(3):e91982.

Fulcher et al., 2014b. Fulcher, B. D., Phillips, A. J., Postnova, S., and Robinson, P. A. (2014b). A physiologically based model of orexinergic stabilization of sleep and wake. *PLoS One*, 9(3):e91982.

Gao et al., 2025. Gao, M. L., Williams, J. P., and Kutz, J. N. (2025). Sparse identification of nonlinear dynamics and koopman operators with shallow recurrent decoder networks.

Hoffstein et al., 1995. Hoffstein, V., Mateika, S., and Hanly, P. (1995). Snoring and arousals: a retrospective analysis. *Sleep*, 18(10):866–872.

Kim et al., 2026. Kim, J. R., Park, H. J., Paik, S. M., Han, S.-K., Lee, W.-J., Yoon, J.-E., Kim, D., Yang, K. I., Chu, M. K., and Yun, C.-H. (2026). Morning sleep inertia and its associated factors: Findings from a nationwide study. *PLoS One*, 21(1):e0337992.

Lacaux et al., 2024. Lacaux, C., Strauss, M., Bekinschtein, T., and Oudiette, D. (2024). Embracing sleep-onset complexity. *Trends in Neurosciences*, pages S0166–2236.

Li et al., 2025. Li, J., Ilina, A., Peach, R., Wei, T., Rhodes, E., Jaramillo, V., Violante, I. R., Barahona, M., Dijk, D.-J., and Grossman, N. (2025). Falling asleep follows a predictable bifurcation dynamic. *Nature Neuroscience*, pages 1–11.

Miller et al., 2009. Miller, K. J., Zanos, S., Fetz, E. E., den Nijs, M., and Ojemann, J. G. (2009). Decoupling the cortical power spectrum reveals real-time representation of individual finger movements in humans. *Journal of Neuroscience*, 29(10):3132–3137.

Ngo et al., 2013. Ngo, H.-V. V., Martinetz, T., Born, J., and Mölle, M. (2013). Auditory closed-loop stimulation of the sleep slow oscillation enhances memory. *Neuron*, 78(3):545–553.

Occhionero et al., 2021. Occhionero, M., Fabbri, M., Tonetti, L., Martoni, M., and Natale, V. (2021). Time course of sleep inertia dissipation in memory tasks. *Applied Sciences*, 11(8):3354.

Roberts et al., 2017. Roberts, J. A., Friston, K. J., and Breakspear, M. (2017). Clinical applications of stochastic dynamic models of the brain, part ii: A review. *Biological psychiatry: cognitive neuroscience and neuroimaging*, 2(3):225–234.

Trotti, 2017. Trotti, L. M. (2017). Waking up is the hardest thing i do all day: Sleep inertia and sleep drunkenness. *Sleep medicine reviews*, 35:76–84.

Vallat et al., 2019. Vallat, R., Meunier, D., Nicolas, A., and Ruby, P. (2019). Hard to wake up? the cerebral correlates of sleep inertia assessed using combined behavioral, eeg and fmri measures. *Neuroimage*, 184:266–278.

Weinhold et al., 2011. Weinhold, S. L., Seeck-Hirschner, M., Nowak, A., GÖder, R., and Baier, P. C. (2011). Wake-rem sleep transitions for measuring rem sleep disturbance: comparison between narcolepsy, idiopathic hypersomnia and healthy controls. *Sleep and Biological Rhythms*, 9(3):172–177.

Yang et al., 2016. Yang, D.-P., McKenzie-Sell, L., Karanjai, A., and Robinson, P. (2016). Wake-sleep transition as a noisy bifurcation. *Physical Review E*, 94(2):022412.

Response to Reviewer #2

General comment

The manuscript presents a significant and novel contribution to the computational modeling of sleep regulation. It moves beyond the traditional view of sleep as an abrupt step-function, proposing a minimally-parameterized stochastic dynamical model that accurately captures the non-monotonic, “flickering” dynamics characteristic of the sleep-onset period (SOP). By modeling the transition as a noisy, slowly tilting bistable potential landscape, the authors provide an elegant framework to estimate two crucial, person-specific parameters: the rate of landscape change (alpha) and the intrinsic noise level (sigma). Most importantly, the study validates the relevance of this mechanistic approach by showing that these estimated dynamic parameters correlate significantly with subjective measures of sleepiness, thereby offering novel, quantitative biomarkers for characterizing individual variability in the complex process of falling asleep. While this study offers a promising theoretical framework of sleep-onset transition, several major and minor issues related to model validation, novelty in light of recent literature, and data interpretation must be fully addressed before the manuscript can be reconsidered for publication. My specific comments are detailed below.

Response. We thank the reviewer for their positive assessment of our work.

Major comments

Comment 1

A Paper published last month (Li, Junheng, Anastasia Ilina, Robert Peach, et al. “Falling Asleep Follows a Predictable Bifurcation Dynamic.” Nature Neuroscience,October 28, 2025, 1–11) also

uses a fold bifurcation model to describe the sleep-onset process. The readers will inevitably compare this paper with the authors' work. The authors must introduce a section, likely in the discussion, that compares their model to other approaches and explains why their model is superior for their task.

R1. We thank the reviewer for drawing attention to the work of [Li et al., 2025]. We summarize here their approach (as we understand it) and clarify how our model differs in aims and assumptions.

1. **Slow/monotonic vs fast/bistable:**
   - **Their work:** Li et al. focus on the final "no-return" tipping point at the end of the SOP, and adopt heavy temporal smoothing (1-min moving-median filter on a 6-s feature updated every 3s), which makes their embedding (sleep distance) almost monotonic, removing much of the bistable jumping.
   - **Our work:** We focus on the full progression during the SOP, at a much finer time resolution (at the scale of milliseconds instead of minutes) without smoothing away fast transitions; we explicitly study the intermittent, bistable changes and model individual variability as distinct parameters of the same dynamical system.

   Using the same subject in Fig. 16, we applied Li et al.'s pipeline to compute the sleep-distance measure, and compare it with to our embedding $\mu(t)$. While both measures roughly capture relatively similar fast dynamics, temporal smoothing in the work of Li et al. (moving median) removes almost all intermittent jumps. Smoothing our embedding $\mu(t)$ with similar parameters yield a similar morphology.

2. **Deterministic vs Stochastic**:
   - **Why a deterministic model is appropriate for their target**: By focusing on the final no-return point and applying heavy temporal smoothing to the sleep-distance trajectory, a deterministic, non-autonomous bifurcation model is a natural choice to work with such a slowly-varying, nearly monotonic signal.
   - **Why we adopt a stochastic framework for the SOP**: In our data at finer temporal resolution, the sleep-onset period often contains intermittent bistable excursions. In fact, this metastability during SOP has been frequently reported in the recent literature [Andrillon et al., 2021, Lacaux et al., 2024, Roberts et al., 2017]. These irregular reversals are a dynamical feature of the transition, which cannot be explained by a fully deterministic model, even with additive observation noise. Instead, these patterns arise naturally and parsimoniously in noise-driven bistable dynamics, where intrinsic fluctuations interact with a slowly changing landscape.

3. **Within-individual stability vs between-individual variability**: [Li et al., 2025] report on two prediction tasks: one tests if the sleep-onset centroid (a similar concept to the sleep mode U_s^1 in our work) is preserved from one night to the next within individual; the other predicts the temporal position of the tipping point from the within-individual embedding. These 2 tasks do not report on the inter-individual variability of the phenomenon, which may be less important than assumed. First, in our work, U_s^1 are in fact quite similar even across different subjects (see Fig.17), so it is perhaps not surprising that they are also preserved across several nights. Second, the standard deviation of within-individual predictions of tipping point is of the same order as the standard deviation of the across-individual distribution (Fig. 18, reproduced from [Li et al., 2025]). This suggests that the paper in fact aims to model a uni-

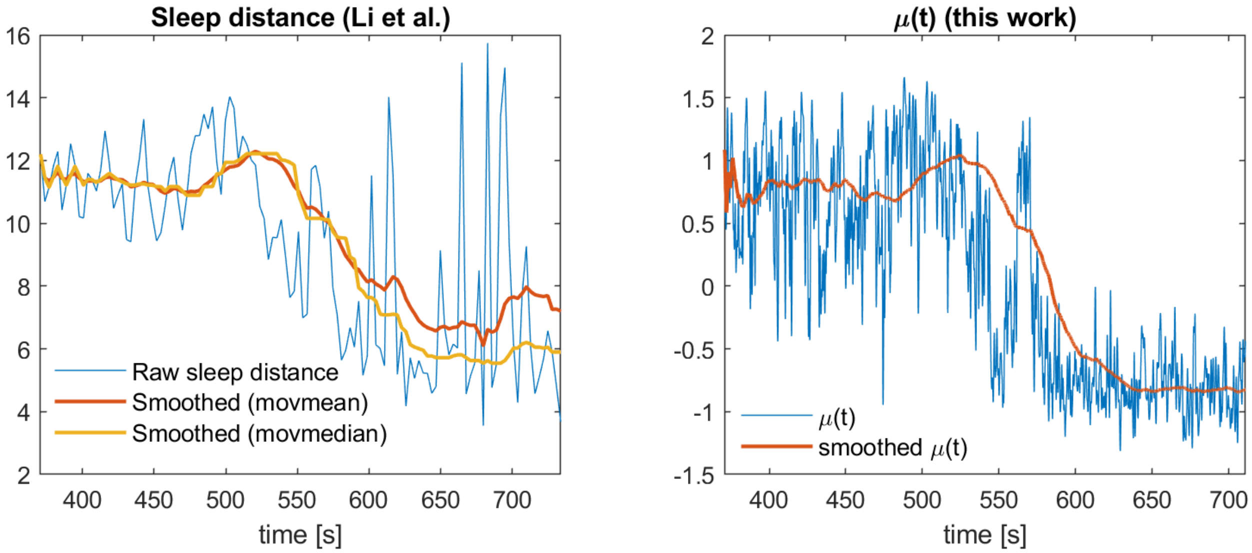

Figure 16: **Comparison of embeddings with [Li et al., 2025]** Left: The sleep distance (Li. et al) is computed as the Euclidean distance between the 49-dimensional feature vector for each 6-s window, and the sleep-onset centroid. The feature set includes power spectrum features, nonlinear features (catch22) and complexity features (e.g., Lempel–Ziv complexity). Before modeling it, Li et al. smooth the time-series with a 1-min moving-median filter (orange). Left: Our embedding, $\mu(t)$, is obtained by projecting the spectrogram bin to the dominant mode difference ($U_w^1 - U_s^1$). When smoothed using the same settings as Li et al. (red), the two curves present the same monotonic morphology.

> versally shared tipping point across individuals, perhaps due to the modeling assumptions of monotony and determinism mentioned above. In contrast, in our work, we explicitly model the intra- and inter-individual variability as distinct parameterization of the underlying model, and attempt to bring light to the meaning of individual parameters by linking them to other sleep variables such as subjective sleepiness.

Taken together, these differences reflect complementary modeling goals rather than a disagreement about whether a bifurcation perspective is useful. Because Li et al. define the phenomenon of interest as the final no-return tipping event and capture the SOP through a strongly smoothed, monotonous sleep-distance trajectory (**point 1**), a deterministic bifurcation model becomes a natural choice for describing a shared group-level signature (**point 2**). A direct consequence of this design is that much of the fast intermittency is averaged out, and intra-/inter-individual differences are primarily discussed as modest dispersion around a common curve. In contrast, we explicitly target the full SOP progression at finer temporal resolution, preserve intermittent bistable excursions, and therefore adopt a stochastic framework in which individual variability is encoded as meaningful differences in model parameters.

Changes. Given the recent prominence of this paper, we have included a dedicated paragraph in the Discussion (**see Line 377 - 406**) as well as introduction (**see Line 42 - 54**) comparing the two approaches and updated our abstract. We have included Fig 16 as **SI Fig. 4**

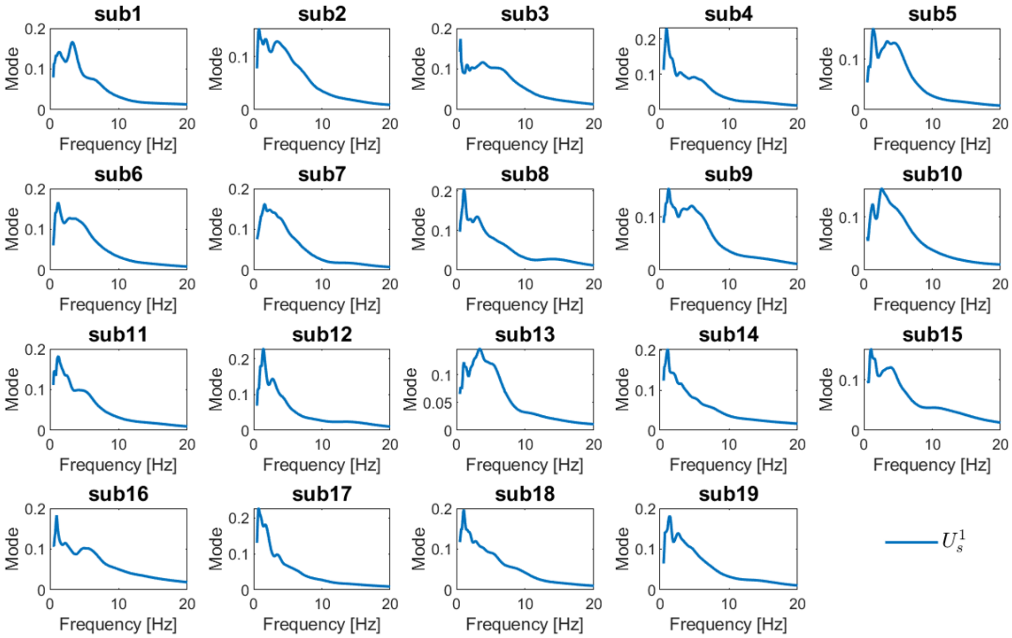

Figure 17: **The first SVD mode of sleep segments across our test dataset.**

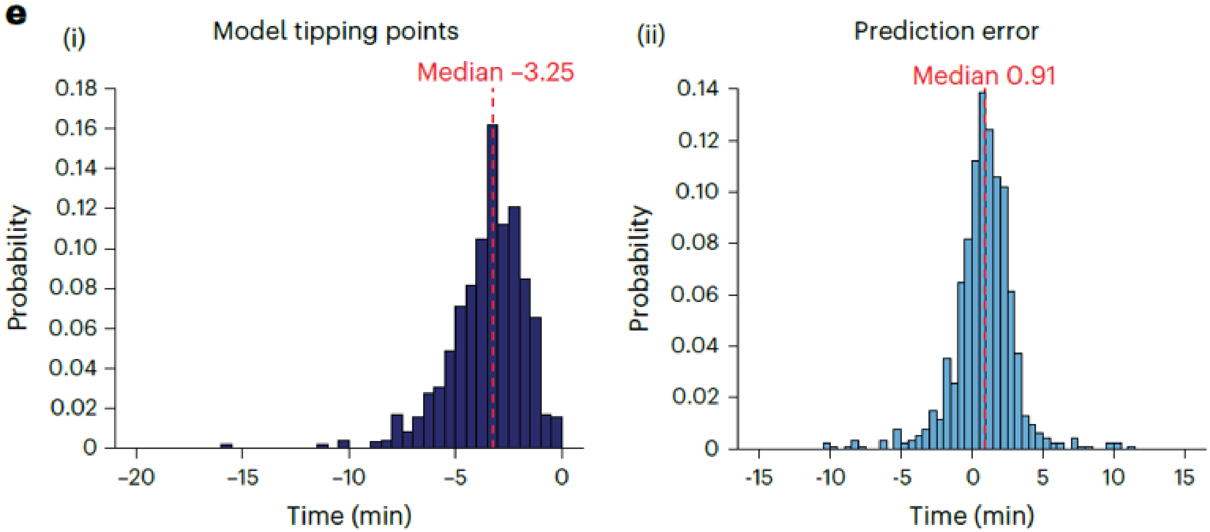

Figure 18: **Tipping point distributions.** These two panels are excerpted from the Fig. 4 of [Li et al., 2025], and show the distributions of tipping pointed computed post-hoc (across-individual) and the tipping point prediction error (within-individual).

Comment 2

The authors mentioned the reduced Philipps-Robinson model (reference #15), which is probably the first model that uses bifurcation to quantify sleep-wake transition. Is the authors' model

mathematically equivalent to the reduced PR model (in terms of the bifurcation structure)? If it does, can authors identify the biological components in the reduced (or the original) PR model that correspond to the parameters (alpha, beta, sigma, etc) in the proposed model and explore what biological processes contribute to "flickering?" The manuscript is submitted to PLoS Computational "biology," hence a bit more discussion in biology is highly appreciated.

R2. We thank the reviewer for pointing to the reduced Phillips–Robinson (PR) model. Our work is directly inspired by this line of research, and in particular by the reference 15 [Yang et al., 2016], which explicitly highlights that future work should incorporate both the changing rate of the control parameter (in our case, α) and the non-negligible variance of the driving noise from experimental data. Below we clarify the mathematical relationship between the reduced PR model and our SOP model, and discuss plausible physiological correlates of our parameters.

Our model is *not* mathematically equivalent to the reduced PR normal form. In [Yang et al., 2016], the full PR dynamics are reduced to a 1-D normal form that can be interpreted as a particle evolving in a cubic potential $U(x) = ax^3 - bx$ with only one stable state and one control parameter $b = b_0(D_c - D)$. This reduction aims to capture the fluctuations close to the critical point, where the wake attractor disappears at $D = D_C$. In contrast, our model uses a quartic double-well potential, which explicitly targets the bistable regime where wake and sleep states coexist. In our framework, the wake-to-sleep transition occurs when the landscape is gradually tilted to lower the barrier between the two basins, and stochastic forcing then triggers switching between them. Hence we are targeting different dynamical regimes.

However, although there are no one-to-one physiological identities, we think that our α and σ play similar roles as parameters in the PR model. The rate parameter (α), defined as local steepness near t_0, plays an analogous role to PR's constant changing rate of control parameter near the fold: $b_0 = -u_1^c/\sigma$, where $u_1 = (V_m - A)/\sigma$ is the dimensionless wake variable (V_m represents mean monoaminergic brainstem nuclei (MA) voltage), and u_1^c is its critical value at the fold. For the noise-level (σ), the PR family of models also provided a concrete interpretation of noise as a physiological ingredient affecting the transition behavior. For instance, [Fulcher et al., 2014b] explicitly discusses that when the threshold for sleep becomes low due to the loss of orexinergic neurons of the lateral hypothalamus (Orx), noise can determine the precise transition timing and produce frequent switching behavior which resembles the phenomenology that we model here.

Changes. We have added a discussion of the relationship between our model and the PR family of models in the introduction (Line 30-41).

Comment 3

The manuscript correctly notes in the Discussion that the 1D SVD-based embedding leverages the core idea of mutual inhibition and that its limitation is missing nuanced amplitude fluctuations. However, the justification for the sufficiency of this reduction relies on a statement that "this 1-D scheme suffices to capture fundamental wake-sleep transitions" without providing the empirical support to back this statement. To rigorously validate the model's foundation, the author must quantify the explained variance of the first SVD mode across the entire cohort. More importantly, the authors should provide evidence that the second (or subsequent) SVD modes do not capture dynamics critical for the wake-sleep transition or that incorporating them does not significantly improve the fit or correlation with the subjective sleepiness scores.

R3. Sorry for the confusion. We do not in fact compute SVD on the total spectrogram of the SOP. Instead, our proposed 1-D embedding $\mu(t)$ is defined by projecting the normalized spectrogram to $U_w^1 - U_s^1$, where the U_w^1 and U_s^1 are the first mode from SVDs performed on the wake and sleep segments separately (see Fig. 19 below for an intuitive illustration). Hence, by construction, $\mu(t)$ is not designed to maximize variance, but to capture how the dominant subspace shifts from the wake states to the sleep states. We agree that the word "suffices" in the context of SVD creates the implication of "proportion of variance explained", and have revised the wording accordingly.

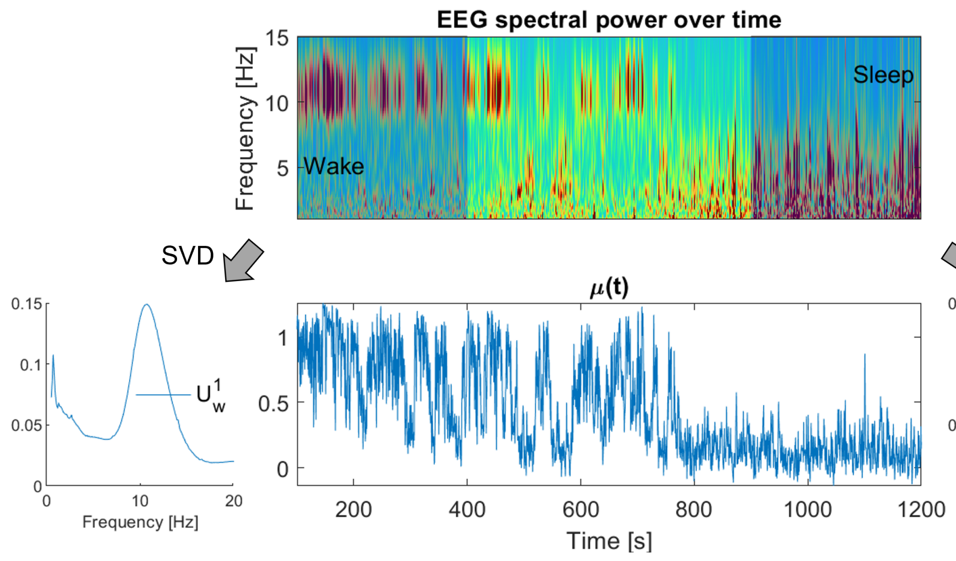

Figure 19: **Illustration of embedding construction.**
We perform SVD separately on the wake and sleep spectrogram segments to obtain the dominant mode U_w^1 and U_s^1, and define $\mu(t)$ as the projection of spectrogram bin onto the direction $U_w^1 - U_s^1$, yielding a 1-D embedding that tracks wake-to-sleep progression.

That being clarified, one may indeed question our use of a single regime-specific dominant mode in each state to construct the embedding. Figure 20 measures explained variance for the first 5 SVD modes of both the wake and sleep segments across the test dataset. Both wake and sleep blocks exhibit a clear **rank-1** structure (2nd singular value wake: $\sigma_w^2 = 0.052 \pm 0.026$; sleep: $\sigma_s^2 = 0.058 \pm 0.022$), consistent with the premise that each regime is well-described by a 1-D spectral subspace.

In addition, to clarify what $\mu(t)$ intends to model, one can compare the spectrogram reconstructed from the $\mu(t)$ embedding (obtained by multiplying the per-timepoint of μ with $U_w^1 - U_s^1$), with a reconstruction using a truncated SVD of the whole spectrogram. The spectrogram SVD is typically rank-2, with a first component V_1^T capturing spectral power, and a second component V_2^T corresponding empirically with $\mu(t)$ (Fig. 1 see also **Reviewer 1 - Comment 5**)). One can see that adding the leading component V_1^T improves spectrogram reconstruction, but it mainly fills in the details of power variability within-state (e.g., large slow-wave bursts in sleep). Our 1-d embedding $\mu(t)$ is constructed to track progression along the sleep-onset period, and in practice that does not correspond to the component that explains maximal variance.

Changes. We have added Figure 20 as supplementary material in the paper (**SI Fg. 1**) and

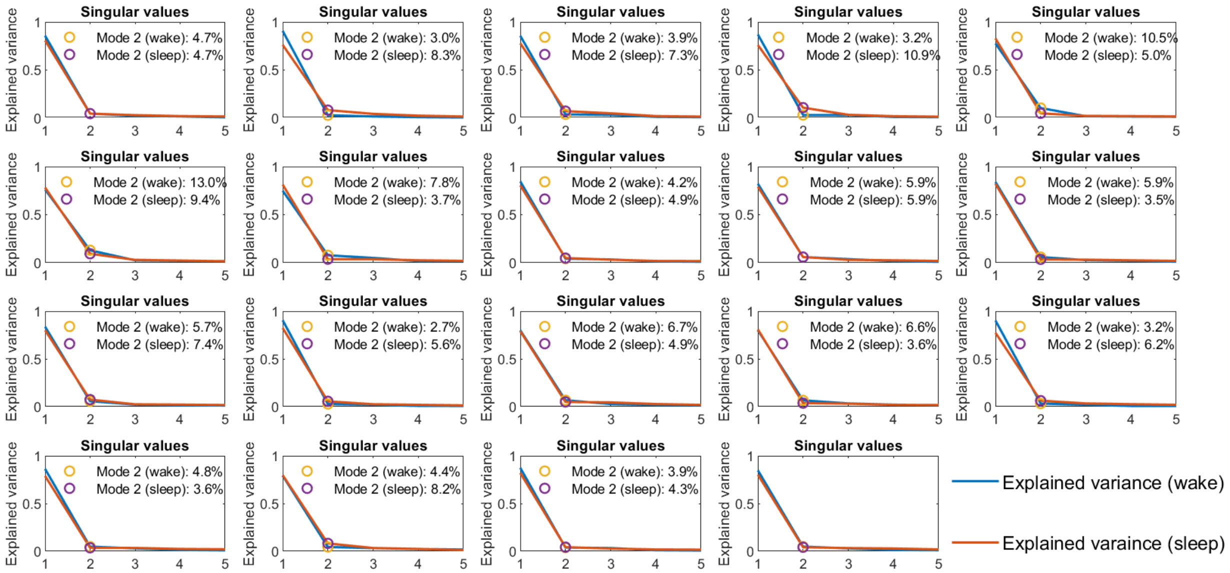

Figure 20: **Explained variance of SVD modes in wake and sleep segments.**
For each participant, we compute the fraction of variance explained by the leading SVD modes separately within the wake (blue) and sleep (orange) spectrogram segments used to construct $\mu(t)$. Across subjects, there is a pronounced rank-1 structure for the wake and sleep segments, with the second mode accounting for only a small additional fraction of variance.

clarified the embedding choice in the discussion (**see line 420 - 444**).

Comment 4

Figure 6c basically shows that the parameter alpha cannot be accurately estimated from individual data. But in Figure 8 left, the authors tried to show that the estimated alpha is correlated with an individual's report of sleepiness. If the alpha values are not accurate, how reliable is the figure 8? I can see (from Fig. 6c) that there are monotonic errors between the estimated and true alpha values. So perhaps the estimated alpha can be corrected by a simple math equation within a certain range? If so, the authors can use the "corrected" alpha values in figure 8.

R4. Thanks for this comment. The saturation of estimated α is an intrinsic property of the model: once $\beta(t) = tanh(\alpha(t - t_0))$ becomes sufficiently steep, increasing α further produces a near step-like drive and the observed trajectory become progressively insensitive to the exact value of α. This saturation phenomenon is illustrated well by simulated spectrograms (Fig. 21 below, reproduced from the paper), where the trajectories in the 2nd row ($\alpha = 0.4$) look very similar to the trajectory in the 3rd row ($\alpha = 1$) for one fixed noise realization. Consistent with this interpretation, we observe a similar nonlinear trend using an independent inference method (simulation-based inference or SBI, see **Comment 6** and Fig. 22 below), supporting that this behavior reflects intrinsic properties of the model as well as reality constraint (**single-observation for a stochastic system**) rather than a specificities of the MCMC algorithm.

There are two reasons why the correlations shown in the paper's Figure 8 are reliable, in our opinion. First, the estimated α over the dataset of participants mostly lie in the pre-saturation

regime (below ~ 0.6), a region where the parameter estimation is reasonably linear. This is physiologically plausible regime for sleep-onset, where the sleep-drive is expected to change gradually rather than as an abrupt step. Second, Figure 8 uses Spearman's rank correlation coefficient (ρ), which depends only on the rank ordering of the estimated parameter values, not their absolute magnitude. As a result, any monotone transformation of the estimated $\hat{\alpha}$, including a nonlinear rescaling would leave Spearman's ρ unchanged and would only rescale the x-axis. Therefore, Figure 8 is reliable with respect to the stated statistical claim linking $\hat{\alpha}$ to behavioral sleepiness.

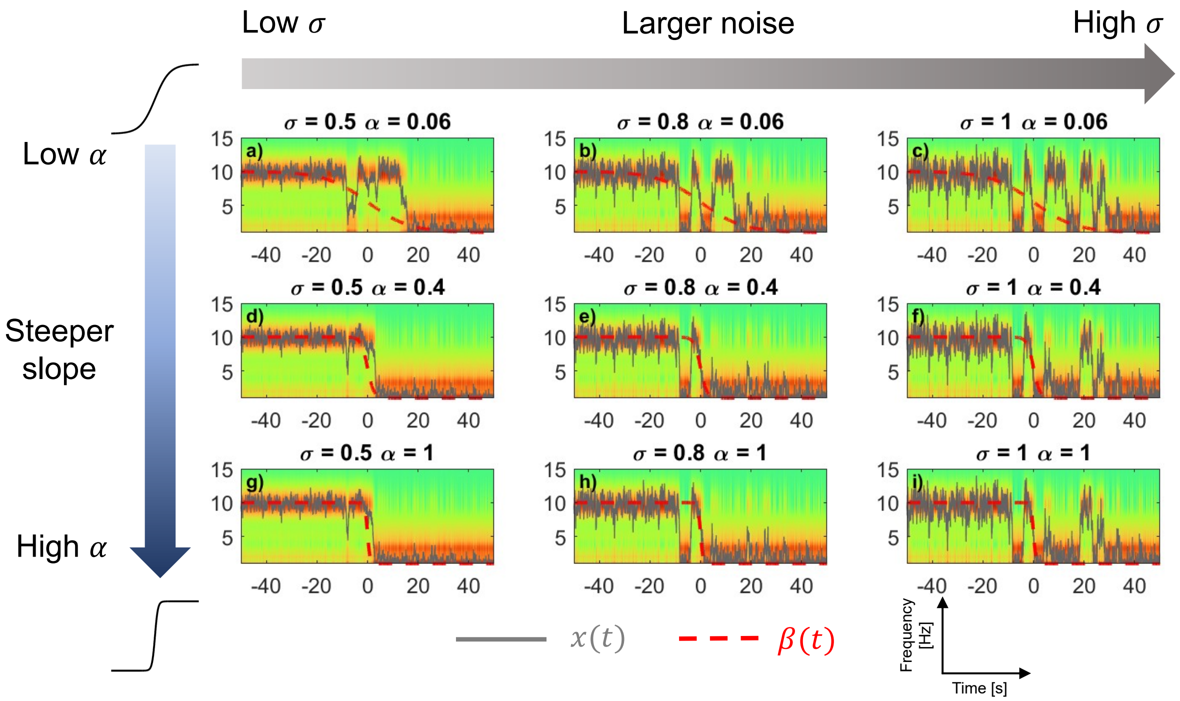

Figure 21: Spectrograms simulated with various values of α and σ, reproduced from the paper's Figure 3

Regarding the reviewer's suggestion of applying a non-linear correction to the estimated α, we think that, in the stochastic setting, the estimator's bias is not a function of α alone but strongly depends on the noise-level as well as particular noise-realization (e.g., under specific noise realization, the trajectory corresponding different α values could look very similar), all contributing to make a single correction equation not well-defined. Hence, rather than imposing a universal correction with additional assumptions, we report $\hat{\alpha}$ directly and assess adequacy using model-checking and predictive diagnostics (e.g., KL divergence, RMSE, likelihood - see **Reviewer 1 - Comment 8**) and switch number (see **Comment 6** below) to confirm that the fitted model reproduces the key features of the observed trajectories.

Changes. We have clarified in the manuscript (**see line 278 - 281**) about the reasons why the correlations in Figure 8 are valid despite the limitations on estimating large alpha values.

Comment 5

Following the last comment, is MCMC the best method to estimate parameters? It is even better if the authors can find a better method to estimate alpha without the post-estimate correction suggested above?

R5. It is indeed a good question. If another estimation method does not create a saturation effect on α, then the conclusion would be that it is MCMC-specific, and incidentally we could replace MCMC with the better method.

Following the reviewer's suggestion, we tried simulation-based inference (SBI, [Deistler et al., 2025]) as a replacement for MCMC to estimate the model parameters. First, we generate a training set of 30 000 simulated trajectories. For each trajectory, rather than using a fixed discretized parameter grid, we independently drew a parameter pair (α, σ) from the uniform priors ($\alpha \sim \mathcal{U}(0.05, 0.6)$, and $\sigma \sim \mathcal{U}(0.2, 1)$), fixed $t_0 = 50$, and simulated the cubic SDE using Euler-Maruyama (dt = 0.01, 8000 steps). We then extracted 56 summary features from each raw trajectory, organized into three groups:

- global features, that describe overall amplitude and variability (e.g., distribution statistics (mean/std), dwell-time distribution, and autocorrelation) over the whole trace.
- transition features, that describe the change around t_0 (e.g., pre/post-t_0 differences, crossing time, and local slope near the change); and
- Pre-escape features, that compute lag-1 dependence and variance measures in sliding windows over $[\tau - H, \tau]$ (τ is the time of first-crossing of a fixed-level after t_0), summarized by their average, their trend as τ is approached, and the difference between late vs early windows.

Then we trained a neural posterior estimator (NPE) in the SBI framework on paired samples $(\theta, s(x))$, learning a conditional density $q_\phi(\theta \mid s)$ that approximates the posterior $p(\theta \mid s)$. Finally, we evaluated parameter recovery by selecting 10,000 simulated cases, sampling the posterior (5000 samples per case) to obtain point estimates of α.

Figure 22 plots estimated α versus true α. The mean estimation shows a monotonic trend but exhibits a nonlinear distortion / saturation similar to that observed with MCMC inference. Hence, the deviation from perfect recovery is not easily fixed by switching to another inference method, and is primarily due to the intrinsic model property.

Changes. Because the SBI evidence is a bit exploratory, we didn't includ it in the revised paper, but we could include it in the revised paper or supplementary material if the reviewer deems it necessary.

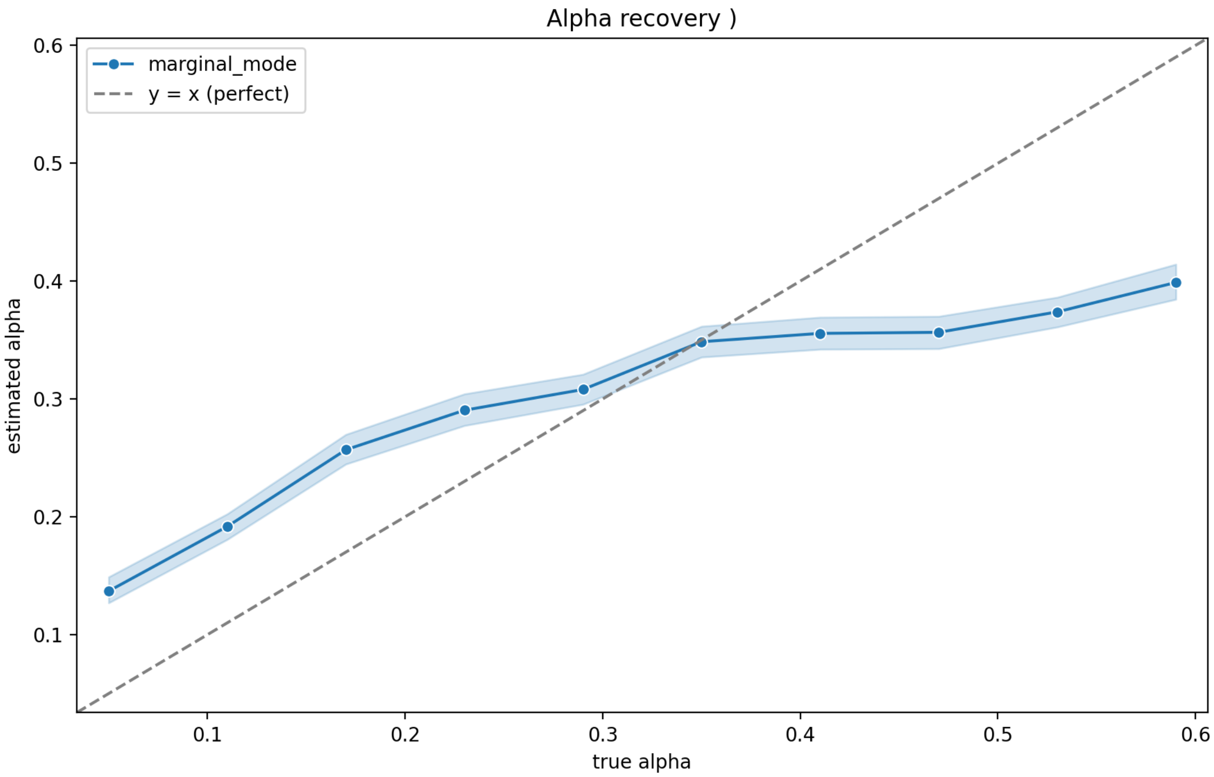

Figure 22: **Parameter α recovery under SBI.** We evaluate the parameter recovery on $N = 10\,000$ simulated test trajectories by drawing posterior samples (5000 per case) and extracting a point estimate $\hat{\alpha}$ by taking its marginal mode. The dashed line shows the ideal relation ($\hat{\alpha} = \alpha$). Orange markers summarize $\hat{\alpha}$ using sliding windows: for each window center (spaced by $\Delta = 0.05$, we include all test cases with $\alpha \in [c - 0.0125,\ c + 0.0125]$. and plotting the mean $\hat{\alpha}$ with error bars indicating confidence interval across cases in that window. The annotation n indicates the number of test case contributing to each window.

Comment 6

Figure 8 right. Why is the noise level (sigma) correlated with reported sleepiness? In the manuscript, the authors explained that a higher sigma can lead to earlier sleep onset. However, a higher sigma may also produce more “sleep-to-wake” transitions, which should not happen for a person with a high sleepiness level. The authors should analyze their simulated and experimental data and examine this issue. Maybe the authors can also explain it from a biological perspective. What biological processes contribute to sigma (see my major comment #2) and whether they make sense.

R6. We appreciate this question. Our claim that α and σ interact in such a way that larger σ may lead to earlier transition to sleep (making it compatible with increased sleepiness) was backed by the illustrative simulations included in the manuscript’s Fig. 3 (reproduced above as Fig 21), but we indeed didn’t provide a systematic analysis of how the two parameters affect the number of switch between states in the model.

To do so, we quantified switches N_{sw} in the simulated model as the number of zero-crossings of the state $x(t)$ and computed switch statistics (mean and standard deviation of N_{sw}) from 100 simulated trajectories across a (α, σ) grid. In the simulated case (Fig. 23-left), the mean number of switches increases with σ (left to right), but this effect is strongly modulated by α. When α is small (slow landscape tilt), the system remains near the bistable/low-barrier regime for longer, allowing stochastic forcing to kick the state back and forth repeatedly, thus producing more switches. When α is larger (faster tilt), the transition window is shorter and the trajectory switches more quickly, limiting the number of back-and-forth transitions even when there is a larger σ. The nonzero standard deviation (Fig. 23-right) across repetitions reflects trial-to-trial variability due to stochastic properties of the system.

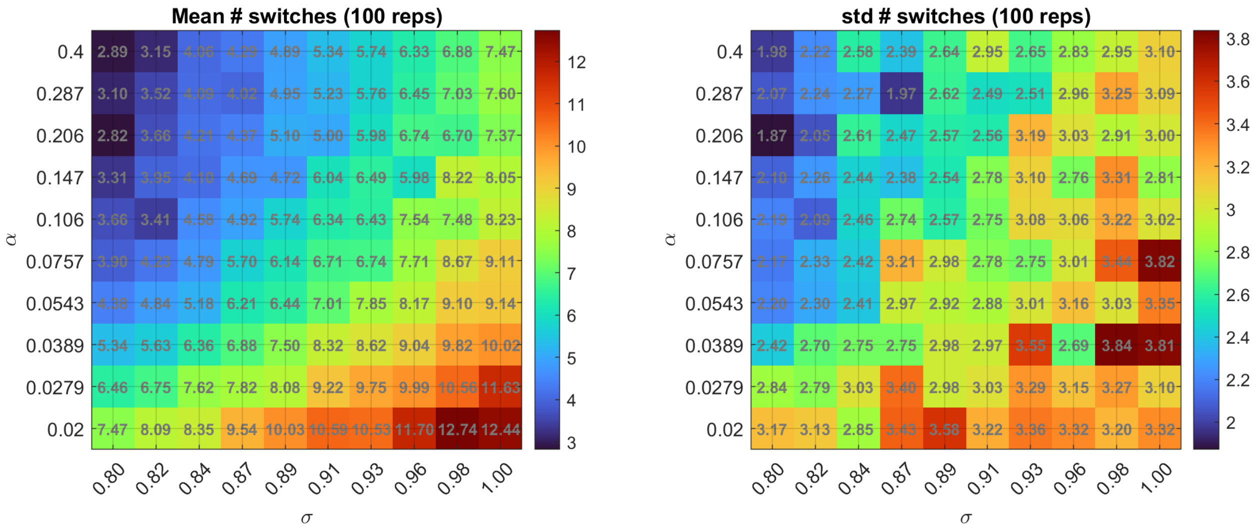

Figure 23: **Switching statistics across the (α, σ) grid (simulated case).** Mean (left) and standard deviation (right) of the number of switches computed from 100 simulated trajectories per parameter pair. For each trajectory, we simulate the cubic SDE with the specified (α, σ). Switches are detected using a robust zero-crossing rule applied to the simulate state $x(t)$. The heatmaps illustrate an interaction between α and σ: increasing σ tends to increase switching while increasing α shortens the bistable window and reduces opportunities for repeated back-and-forth switches. The non-zero standard deviation reflects trial-to-trial variability inherent to stochastic dynamics.

To extend this analysis to the real-data case, we applied the same zero-crossing switch detector to each subject's embedding ($\mu(t)$), and map them with their estimated $\hat{\alpha}$ and $\hat{\sigma}$. Fig. 24-left shows that, consistent the simulated case, similar switch counts can arise from relatively different (α, σ) combinations. For example, sub15 with small $\hat{\alpha}$ exhibits relatively frequent switching despite having the lowest $\hat{\sigma}$, which is consistent the model prediction that slow landscape tilt can increase the time spent near the transition region and thereby increases the opportunities of noise-driven back-and-forth switching. However, because switching is stochastic and we only have one realization for each subject, we do not expect one-to-one agreement in switch counts for individual subjects. To quantify whether our model estimation on real-data is able to preserve the expected switch statistics of the underlying model, we simulated 100 trajectories per subject using that subject's fitted parameters, and compared the average number of switches over these simulations with the actual number of switches in the subject's SOP. There was a positive statistical association between real and simulated switching (Pearson $r = 0.51, p = 0.036$; Fig. 24-right), which supports the idea that

the fitted model correctly captures cross-subject variability in switching.

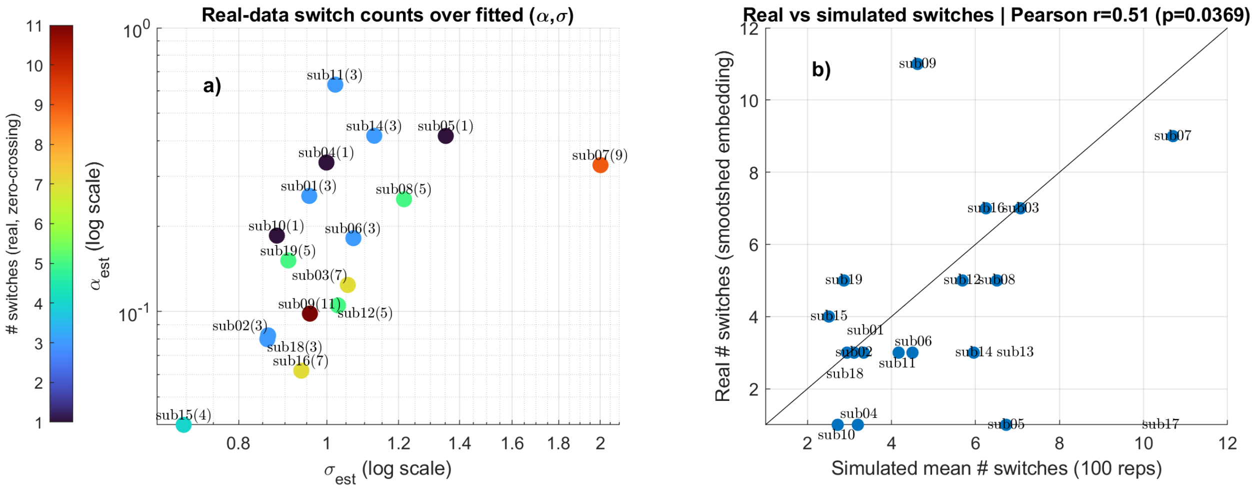

Figure 24: **Switch counts from real data and simulations using fitted (α,t_0,σ).** (a) Each point is a subject placed at their estimated ($\hat{\alpha}$,$\hat{\sigma}$) coordinates. The label subxx(n) reports the switch count computed from the smoothed embedding $\mu(t)$ using a zero-crossing switch detector. (b) Real switch counts versus simulated switch counts obtained by averaging over 100 simulated trajectories per subject using that subject's fitted parameters. Solid line: $y = x$ line.

In sum, while the reviewer is correct that increased σ may in general lead to more numerous switches, including more backward sleep-to-wake transitions (which seems counter-intuitive), the simultaneous increase of α in the context of high sleepiness contributes to counter-balance the number of switches, down to a comparable level as what it is with low α and low σ (top right corner vs bottom left corner in Fig. 23-left).

Changes. We have added this systematic analysis of the model's behavior in terms of switches in several sections of the manuscript : first, we have added Fig. 23 to complement the illustrative example of Fig.3; second, we have added Fig. 24-right to the validation section of the paper to show that, beyond goodness of fit (RMSE, KL and, following **Reviewer 1 - Comment 8**, log-likelihood), our model estimation preserves the switching phenomenology of the data; finally, we have discussed the correlation results with subjective sleepiness by referring back to Fig. 23-left (**see line 299 - 310**).

Minor comments

Comment 7

Line 35. "saddle-point bifurcation" should be "saddle-node bifurcation."

R7. We have replaced accordingly.

Comment 8

Figure 2a. I do not see "dashed lines" mentioned in the main text and figure legends.

R8. Sorry for the mistake, we have put the dashed line back in Figure 2.

Comment 9

Model equations are repetitively shown in Results and Methods. The authors should just choose one place (preferably in the Methods) to show and explain.

R9. We have changed this in the revised manuscript and only put in the Methods section.

Comment 10

Line 109 (eq 1). Replace x' with dx/dt for clarity.

R10. We have replaced x' with $\frac{dx}{dt}$ for eq.1.

Comment 11

Figure 5 right. Why did the authors only test three sigma values? Can they test more sigma values, similar to the alpha values in Figure 5 left, to make the trends of the curve clearer?

R11. Thank you for this comment. We have added more sigma values to produce a clear trend of the σ estimation to replace the original Figure 6. The new figure is below (Fig. 25).

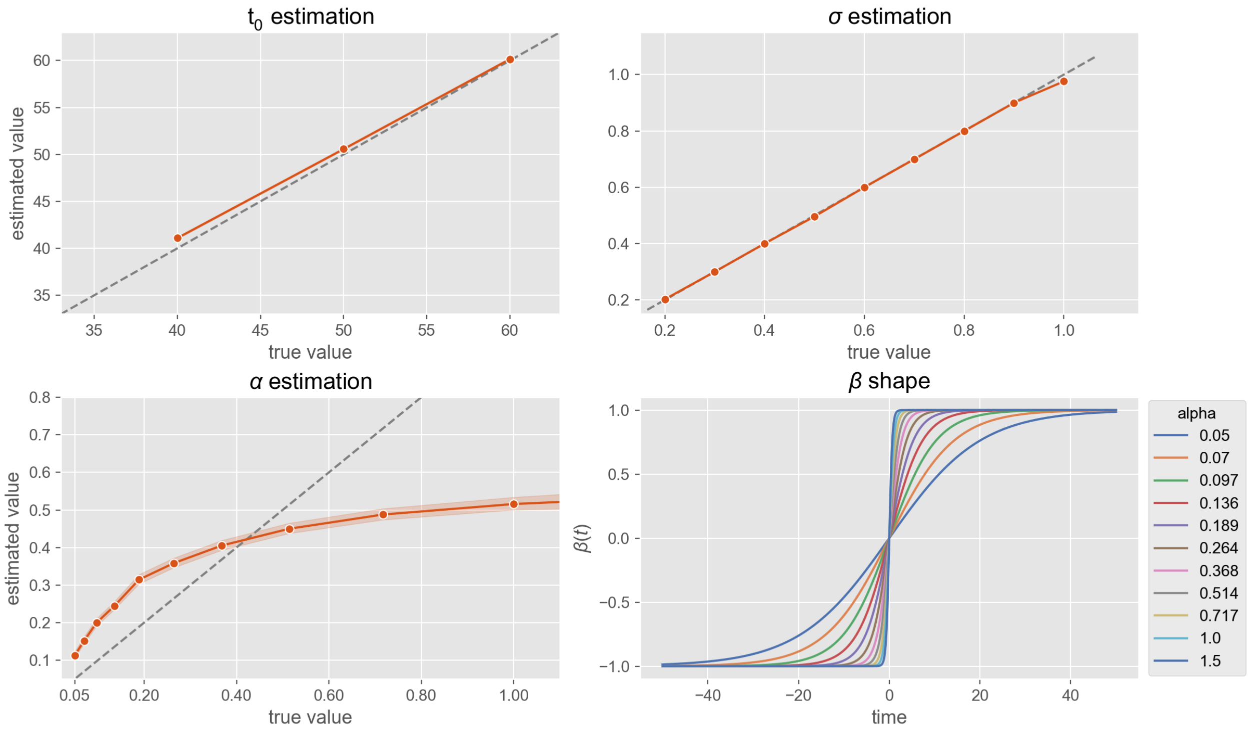

Figure 25: **Replacement for Fig.6, using linear x-axes and a larger range of σ values.**

Comment 12

Figure 4 right and Figure 5b & 5c. The plots used to compare estimated and true parameter values (specifically for alpha in Fig. 5C) are confusing and misleading due to the use of a nonlinear x-axis scale. To rigorously demonstrate the accuracy of the parameter estimation procedure, the author must replot these data using linear x and y axes with the same range. This should include a reference 45 degree line (y=x) to allow readers to visually and quantitatively assess the magnitude of the estimation error (i.e. how "off" the estimated values are from the true values).

R12. We have replaced the categorical axis with numerical axis to make the plots linearly spaced (Fig. 25).

Changes.

Comment 13

Line 396. Were all EEG channels used when the authors applied SVD analysis? More details should be given about the SVD analysis.

R13. In the original manuscript (Line 375), we mentioned only the central-occipital electrode (Oz) was selected. The rationale is that a prominent occipital alpha rhythm (8–12 Hz) during wake is a prerequisite for producing a near rank-1 structure in the wake-state spectrum: when alpha dominates the spectral energy, the leading singular mode captures this single dominant component robustly and consistently across time. This property is essential for our construction of $\mu(t)$, which is designed to track how the dominant spectral subspace shifts from wake to sleep (i.e., from an alpha-dominated mode to a low-frequency-dominated mode). Using Oz heuristically maximizes the alpha-band SNR and therefore yields a stable reference dominant mode for $\mu(t)$, whereas pooling across heterogeneous channels could dilute the alpha dominance and weaken the rank-1 structure that our subspace-tracking relies on.

Changes. We have emphasized these details in the embedding extraction section to make it clear (**see line 566**).

References

Andrillon et al., 2021. Andrillon, T., Burns, A., Mackay, T., Windt, J., and Tsuchiya, N. (2021). Predicting lapses of attention with sleep-like slow waves. *Nature communications*, 12(1):3657.

Basner and McGuire, 2018. Basner, M. and McGuire, S. (2018). Who environmental noise guidelines for the european region: a systematic review on environmental noise and effects on sleep. *International journal of environmental research and public health*, 15(3):519.

Buus et al., . Buus, R. M., Genovese, S., and Jespersen, K. V. The art of sleep: examining sleep strategies in the general population with a focus on the use of music for sleep. *Journal of Sleep Research*, page e70006.

Champion et al., 2019. Champion, K., Lusch, B., Kutz, J. N., and Brunton, S. L. (2019). Data-driven discovery of coordinates and governing equations. *Proceedings of the National Academy of Sciences*, 116(45):22445–22451.

Craver, 2007. Craver, C. F. (2007). *Explaining the brain: Mechanisms and the mosaic unity of neuroscience*. Clarendon Press.

Deistler et al., 2025. Deistler, M., Boelts, J., Steinbach, P., Moss, G., Moreau, T., Gloeckler, M., Rodrigues, P. L., Linhart, J., Lappalainen, J. K., Miller, B. K., et al. (2025). Simulation-based inference: A practical guide. *arXiv preprint arXiv:2508.12939*.

Fulcher et al., 2014a. Fulcher, B. D., Phillips, A. J., Postnova, S., and Robinson, P. A. (2014a). A physiologically based model of orexinergic stabilization of sleep and wake. *PLoS One*, 9(3):e91982.

Fulcher et al., 2014b. Fulcher, B. D., Phillips, A. J., Postnova, S., and Robinson, P. A. (2014b). A physiologically based model of orexinergic stabilization of sleep and wake. *PLoS One*, 9(3):e91982.

Gao et al., 2025. Gao, M. L., Williams, J. P., and Kutz, J. N. (2025). Sparse identification of nonlinear dynamics and koopman operators with shallow recurrent decoder networks.

Hoffstein et al., 1995. Hoffstein, V., Mateika, S., and Hanly, P. (1995). Snoring and arousals: a retrospective analysis. *Sleep*, 18(10):866–872.

Kim et al., 2026. Kim, J. R., Park, H. J., Paik, S. M., Han, S.-K., Lee, W.-J., Yoon, J.-E., Kim, D., Yang, K. I., Chu, M. K., and Yun, C.-H. (2026). Morning sleep inertia and its associated factors: Findings from a nationwide study. *PLoS One*, 21(1):e0337992.

Lacaux et al., 2024. Lacaux, C., Strauss, M., Bekinschtein, T., and Oudiette, D. (2024). Embracing sleep-onset complexity. *Trends in Neurosciences*, pages S0166–2236.

Li et al., 2025. Li, J., Ilina, A., Peach, R., Wei, T., Rhodes, E., Jaramillo, V., Violante, I. R., Barahona, M., Dijk, D.-J., and Grossman, N. (2025). Falling asleep follows a predictable bifurcation dynamic. *Nature Neuroscience*, pages 1–11.

Miller et al., 2009. Miller, K. J., Zanos, S., Fetz, E. E., den Nijs, M., and Ojemann, J. G. (2009). Decoupling the cortical power spectrum reveals real-time representation of individual finger movements in humans. *Journal of Neuroscience*, 29(10):3132–3137.

Ngo et al., 2013. Ngo, H.-V. V., Martinetz, T., Born, J., and Mölle, M. (2013). Auditory closed-loop stimulation of the sleep slow oscillation enhances memory. *Neuron*, 78(3):545–553.

Occhionero et al., 2021. Occhionero, M., Fabbri, M., Tonetti, L., Martoni, M., and Natale, V. (2021). Time course of sleep inertia dissipation in memory tasks. *Applied Sciences*, 11(8):3354.

Roberts et al., 2017. Roberts, J. A., Friston, K. J., and Breakspear, M. (2017). Clinical applications of stochastic dynamic models of the brain, part ii: A review. *Biological psychiatry: cognitive neuroscience and neuroimaging*, 2(3):225–234.

Trotti, 2017. Trotti, L. M. (2017). Waking up is the hardest thing i do all day: Sleep inertia and sleep drunkenness. *Sleep medicine reviews*, 35:76–84.

Vallat et al., 2019. Vallat, R., Meunier, D., Nicolas, A., and Ruby, P. (2019). Hard to wake up? the cerebral correlates of sleep inertia assessed using combined behavioral, eeg and fmri measures. *Neuroimage*, 184:266–278.

Weinhold et al., 2011. Weinhold, S. L., Seeck-Hirschner, M., Nowak, A., GÖder, R., and Baier, P. C. (2011). Wake-rem sleep transitions for measuring rem sleep disturbance: comparison between narcolepsy, idiopathic hypersomnia and healthy controls. *Sleep and Biological Rhythms*, 9(3):172–177.

Yang et al., 2016. Yang, D.-P., McKenzie-Sell, L., Karanjai, A., and Robinson, P. (2016). Wake-sleep transition as a noisy bifurcation. *Physical Review E*, 94(2):022412.
