# Supplementary material for: Learning the bistable cortical dynamics of the sleep-onset period
Source: PLoS Comput Biol. 2026 Apr 30;22(4):e1014246. doi: 10.1371/journal.pcbi.1014246 (PMC13152216; doi:10.1371/journal.pcbi.1014246)
Supplement: Attachment — Submitted filename: Response_to_reviewers_auresp_2.pdf [file pcbi.1014246.s004.pdf]

Response to Reviewers

Journal: PLOS Computational Biology

Manuscript title: *[Learning the bistable cortical dynamics of the sleep-onset period]*

Manuscript ID: [PCOMPBIOL-D-25-01784R2]

Corresponding author: [Zhenxing Hu]

Date: [08/04/2026]

Revision: [R2]

Response to Reviewer #1

General comment

Many thanks to the authors for constructively engaging with my comments and a comprehensive revision. I especially appreciated the new results with SINDY, the factored likelihood model, and the parameter degeneracy analysis, as well as the clarified contribution in general and relative to the new Li et al. 2025 paper.

The authors have addressed all my concerns adequately and I think this is a fine and interesting piece of work on the role of potential neural noise mechanisms for sleep onset.

Response. We thank the reviewer for their positive assessment and constructive suggestions, which have helped improve the quality of the paper.

Response to Reviewer #2

General comment

I appreciate the authors' thorough efforts in addressing my previous comments. The additional data and plots significantly strengthen their arguments, and I have no further major concerns regarding the scientific content.

However, upon reviewing the revised figures, I identified several inconsistencies and formatting issues that need to be addressed before publication. I recommend a careful final check of all figures and legends. My specific observations are as follows:

Response. We thank the reviewer for their positive assessment of our work and have addressed the reviewer's minor comments below.

Minor comments

Comment 1

Figure 3: The panel labels "a)" and "b)" are missing.

R1. We have re-marked the label "a)" and "b)" in the figure 3 in the prominent place which is also put as Figure 1 here.

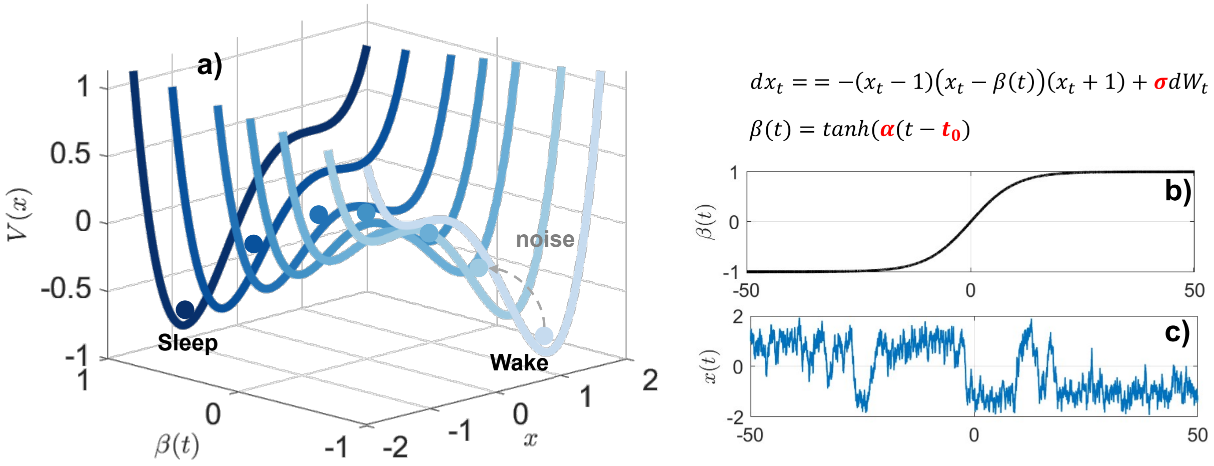

Figure 1: **Schematics of the time-varying stochastic bistable model.**
a) The potential functions, $V(x)$, transitions from a “wake” basin (right) to a “sleep” basin (left) as β shifts from -1 to +1. b) $\beta(t) = tanh(\alpha(t - t_0))$ governs the gradual tilt of the landscape over time. c) A representative trajectory $x(t)$ simulated from the stochastic differential equation Eq. **??**).

Comment 2

Figure 6 (Right): Regarding my previous comment about the ”limited sample size” (formerly Fig 5), the authors acknowledged the issue but updated Fig 7B instead. The specific panel I mentioned (now Figure 6 right) remains unchanged with only three data points. Please ensure this is addressed.

R2. Thanks for this comment. We have added more tested σ values in the Figure 6 in the latest revised version, which is also put as Figure 2 here.

Comment 3

Figure 7: Panel labels ”A–D” are missing from the graphics.

R3. Sorry for the mistake. We have added ”a),b),c),d)” as panel labels in the Figure 7, which is also put as Figure 3 here.

Comment 4

Figure 7 caption: There is a typographical error in the caption where ”Panel C” is cited twice; one of these references should be changed to Panel “B.”

R4. Sorry for the mistake. We have referred it to panel ”b)”, as we switched to the lowercase letters for labeling the figures.

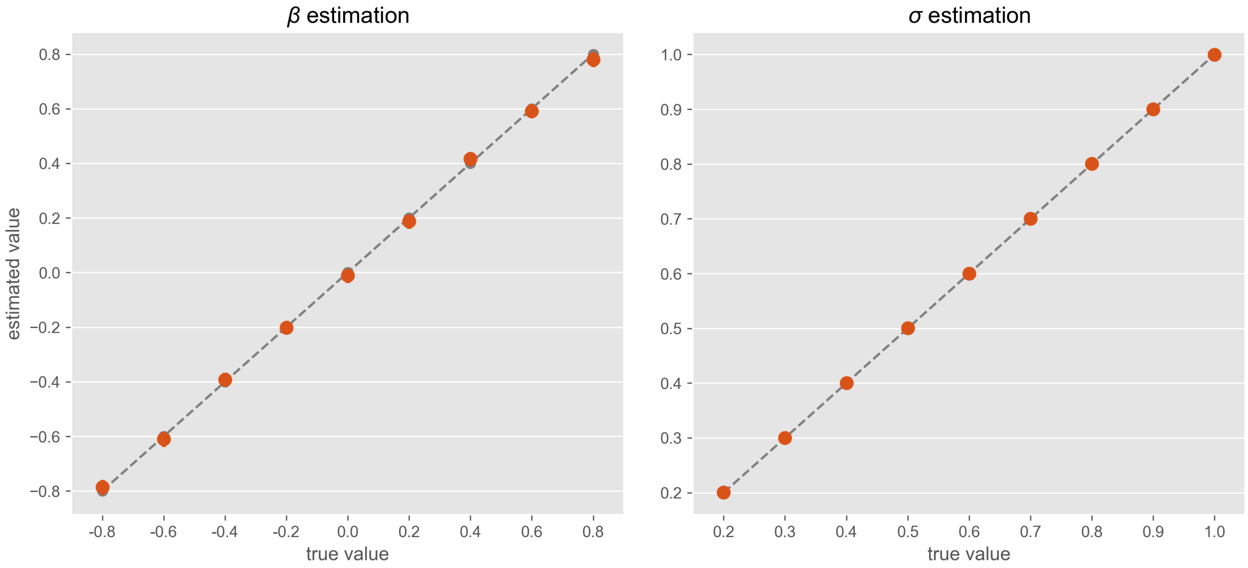

Figure 2: **Model parameter estimation for simulated single trajectories under the stationary landscape assumption ($\beta(t) = \beta$). Left:** 30 trajectories were simulated for every value of $\beta \in \{-0.8, -0.6, -0.4, -0.2, 0, 0.2, 0.4, 0.6, 0.8\}$. Distributions of MCMC estimates for β (y-axis) are plotted against the true values (x-axis). Error bars indicate the 95% confidence interval of the mean estimates across multiple trajectories of the same β value. **Right:** 30 trajectories were simulated for every $\sigma \in \{0.2, 0.3, 0.4, 0.5, 0.6, 0.7, 0.8, 0.9, 1.0\}$. Distributions of MCMC estimates for σ (y-axis) are plotted against the true values (x-axis). Error bars are barely visible across multiple trajectories of the same σ.

Comment 5

Figure 7, Panel B: There is a discrepancy between the plot and the caption. The plot displays data for nine sigma values, whereas the caption only specifies three.

R5. Sorry for the mistake. We have modified the caption to nine σ values and also the main text to make it consistent to the figure.

Comment 6

Consistency in Labeling: There is an inconsistency in the panel labeling style throughout the manuscript. Some figures use lowercase letters (e.g., a, b) while others use uppercase (e.g., A, B). Please ensure a consistent labeling format is applied to all figures.

R6. Thanks for the comment. We have adopted lowercase letters (e.g., ”a)”,”b)”) for the figure labeling to all figures.

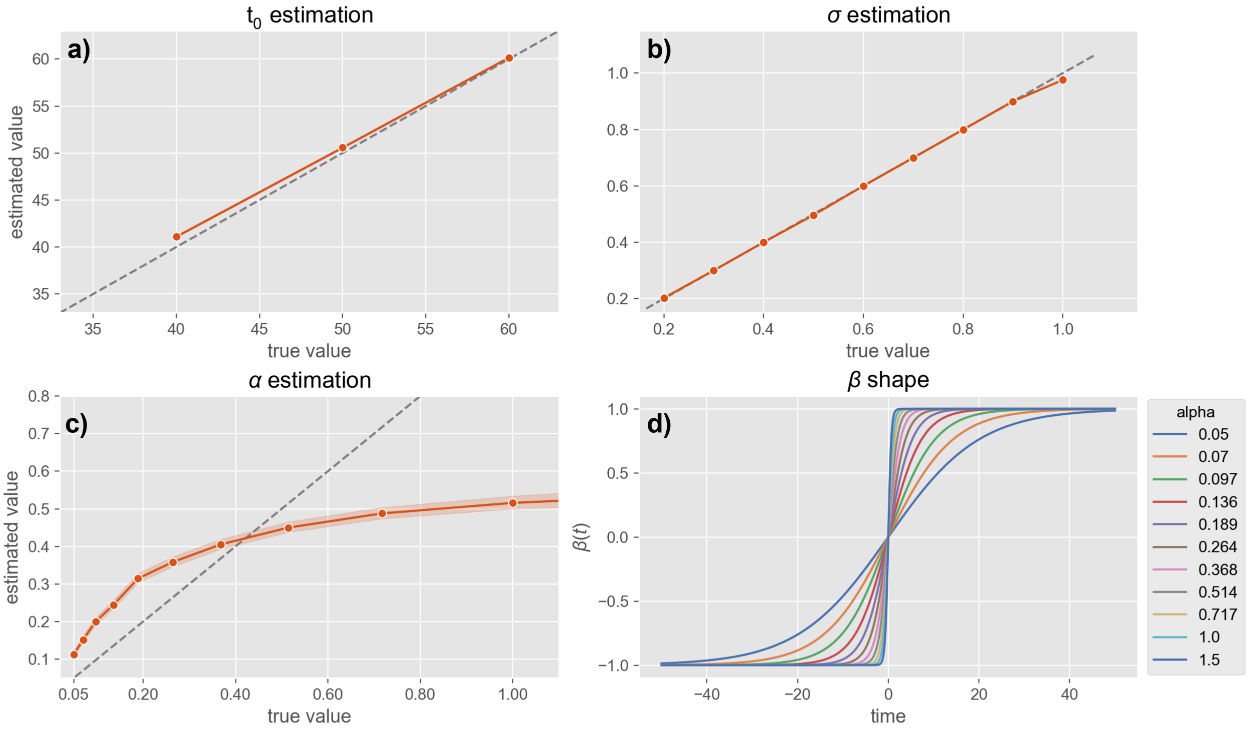

Figure 3: **Model parameter estimation for simulated single trajectories under the varying-landscape, non-stationary assumption.** **a):** 30 trajectories were simulated for every value of $t_0 \in \{40, 50, 60\}$ and recovered MCMC estimates for t_0 (y-axis) are plotted against true values (x-axis). Error bars (barely visible) indicate confidence interval across multiple realizations of the same t_0, and dashed line indicates perfect estimation. **b).** 30 trajectories were simulated for every value of $\sigma \in \{0.2, 0.3, 0.4, 0.5, 0.6, 0.7, 0.8, 0.9, 1.0\}$, and MCMC estimates (y-axis) are plotted against true values (x-axis). Error bars (barely visible) indicate the 95% confidence interval of the mean estimates across the multiple realizations of the same σ, and dashed line indicates perfect estimation. **c):** 30 trajectories were simulated for every value of $\alpha \in [0.05, 1.5]$ (logarithmically-scaled), and MCMC estimates (y-axis) are plotted against true values (x-axis). Error bars indicate the 95% confidence interval of the mean estimates across multiple realizations of the same α, and dashed curve indicates perfect estimation. **d):** Varying $\beta(t)$ shape (x-axis:time in units of model integration instead of seconds) as a function of slope α, illustrating a saturation effect at larger α values.
